# Evolving trends and burden of iron deficiency among children, 1990–2019: a systematic analysis for the global burden of disease study 2019

**DOI:** 10.3389/fnut.2023.1275291

**Published:** 2023-12-07

**Authors:** Dan Long, Chenhan Mao, Yaxuan Liu, Tao Zhou, Yin Xu, Ying Zhu

**Affiliations:** ^1^The First Hospital of Hunan University of Chinese Medicine, Changsha, Hunan, China; ^2^Affiliated Hospital of Integrated Traditional Chinese and Western Medicine, Nanjing University of Chinese Medicine, Nanjing, Jiangsu, China

**Keywords:** iron deficiency, children, global burden of disease, Joinpoint regression model, epidemiology

## Abstract

**Objectives:**

We aimed to provide a timely, comprehensive, and reliable assessment of the burden of iron deficiency (ID) in children between 1990 and 2019 at the global, regional, and national levels to inform policymakers in developing locally appropriate health policies.

**Methods:**

Data related to ID among children younger than 15 years old were analyzed by sex, age, year, socio-demographic index (SDI), and location according to the Global Burden of Disease Study 2019 (GBD 2019). Age-standardized rates were used to compare the burden between different regions and countries. Furthermore, the Joinpoint regression model was used to assess temporal trends from 1990 to 2019.

**Results:**

In 2019, the number of prevalent cases and disability-adjusted life years (DALYs) for ID in children were 391,491,699 and 13,620,231, respectively. The global age-standardized prevalence and DALY rates for childhood ID in 2019 were 20,146.35 (95% confidence interval: 19,407.85 to 20,888.54) and 698.90 (466.54 to 1015.31) per 100,000, respectively. Over the past 30 years, the global prevalence of ID among children has been highest in low-SDI regions, particularly in Western Sub-Saharan Africa, South Asia, and Eastern Sub-Saharan Africa. Since 1990, the prevalence and DALY of ID in children have been declining in most geographic regions. Nationally, Ecuador, China, and Chile have shown the most significant decreases in prevalence. The greatest decline in age-standardized DALY rate was observed in Ecuador, while Burkina Faso experienced the highest increase. Bhutan had the highest prevalence and DALY rates in 2019. On the age level, the prevalence was relatively higher among the <5 years age group. At the gender dimension, the prevalence of ID in children overall was more pronounced in girls than in boys, as was the case for DALY.

**Conclusion:**

Although the burden of ID in children has been declining, this disease remains a major public health problem, especially in countries with low SDI. Children younger than 5 years of age are an important group for whom targeted measures are needed to reduce the burden of ID.

## Introduction

Iron is the most abundant micronutrient in the body and is involved in numerous physiological processes. Iron balance is critical for maintaining organic health (1, 2). The Sustainable Development Goals (SDGs) released by the United Nations General Assembly in 2015 aim to eliminate all forms of malnutrition by 2030. Iron deficiency (ID) affects billions of people around the world and remains the leading cause of anemia, with profound negative impacts on health (3). According to the Global Burden of Disease (GBD) study 2019, the prevalence of ID varied widely across 204 countries and territories, ranging from 1,990.9 to 32,085.7 per 100,000. ID have an adverse impact on human behavior. Children and adolescents with ID often exhibit sluggishness, poor concentration, and reduced learning ability and memory (4, 5). Iron is also involved in thermoregulation and energy metabolism in the body (6, 7). Additionally, ID leads to a substantial reduction in neutrophils and affects their effector functions, which affects the defense ability and makes it more susceptible to infectious diseases (8).

Unfortunately, children’s health has been largely neglected in global public health, as this age group is usually considered healthy. Actually, ID is not only one of the major contributors to the global burden of disease, but it is also the most common nutritional disorder in childhood (9–11). Obviously, children are a key group for ID prevention and treatment (12). However, the prevalence of ID in children varies widely across regions and countries with different economic levels. To our knowledge, no long-term global trends in the epidemiology of childhood ID have been reported. Here, we aimed to comprehensively assess the ID burden in children, including prevalence and DALYs, at global, regional, and national levels.

The GBD Study 2019 is a large database of multinational collaborative research that covers all WHO member countries. It provides a comprehensive assessment of health losses globally between 1990 and 2019 for 204 countries and territories, 369 diseases and injuries, and 87 risk factors (13, 14). It is currently the most extensive and credible database on the burden of disease worldwide and has been widely used in disease burden studies (14, 15). Therefore, in this study, data on the burden of childhood ID, derived from the GBD database, are comprehensive and reliable. In addition, we evaluated temporal trends over the past 30 years. This study will help us to better understand the epidemiology of childhood ID in different geographic regions, so as to formulate appropriate prevention strategies and rationalize the allocation of health resources.

## Materials and methods

### Data sources

Children under 15 years old were included in our analysis. All data for this study were obtained from the GBD study 2019 database (16). The search was done through the official website of IHME^1^ utilizing the GBD result tool. ID in the GBD cause analysis was defined as inadequate iron to meet the body’s needs due to inadequate dietary intake of iron, but not due to other causes of absolute or functional ID (16). ID was mapped to the GBD cause list with International Statistical Classification of Diseases and Related Health Problems, Tenth Revision (ICD10) codes D50–D50.9. Detailed descriptions of the raw data and general methods of the GBD 2019 study have been described in previous publications (16, 17). In this study, data stratified by sex and age group (<5, 5–9, 10–14) were collected from the GBD database from 1990 to 2019 at global, regional, and national levels. Firstly, this study focused on the analysis of prevalent cases, age-standardized prevalence rate (ASPR), DALYs, and age-standardized DALY rate for childhood ID globally, in GBD regions, and in 204 countries and territories (18, 19). Secondly, we also analyzed the relationship between SDI and ID burden in children. The SDI ranges from 0 to 1. A location with an SDI of 0 would have a theoretical minimum level of socio-demographic development relevant to health outcomes, and a location with an SDI of 1 would have a theoretical maximum level of socio-demographic development relevant to these health outcomes. Based on the SDI quintiles, countries and territories in this study were categorized into five groups in ascending order: low SDI (0–0.45), low-middle SDI (0.45–0.61), middle SDI (0.61–0.69), high-middle SDI (0.69–0.81), and high SDI (0.81–1) (20).

### Joinpoint regression analysis

To assess temporal trends in global prevalence and DALY rates between 1990 and 2019, we performed Joinpoint regression analyses (21). The Joinpoint software (version 4.8.01; National Cancer Institute, Rockville, MD, US) was used to understand temporal trends in a structured way and to test which trends between joinpoints were statistically significant (22). A maximum of six line segments (five joinpoints) were applied in the model. Average Annual Percentage Change (AAPC), Annual Percentage Change (APC), and corresponding 95% confidence intervals (CIs) were calculated for this study.

Moreover, R 4.3.1 was used for data analysis and plotting in this study. A *p*-value of less than 0.05 was considered statistically significant.

## Results

### Global level

**Figure 1:**
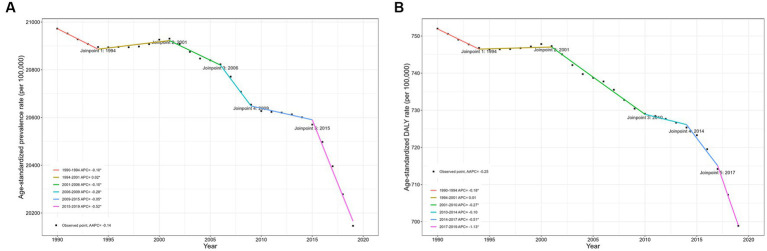
Joinpoint regression analysis of global ID prevalence and DALY rates in children younger than 15 years old from 1990 to 2019. **(A)** Prevalence; **(B)** DALY rates. DALY, disability-adjusted life year; ID, iron deficiency.

In 2019, the number of prevalent cases and DALYs for ID in children were 391,491,699 (95% UI: 382,834,301 to 400,499,411) and 13,620,231 (9,174,234 to 19,794,595), respectively (Tables 1, 2). Compared to 1990, prevalence cases and DALYs in 2019 increased by 5.52 and 2.68%, respectively. The global ASPR and age-standardized DALY rate of ID among children have decreased over the past 30 years. More specifically, the ASPR in 1990 was 20,972.41 (95% CI: 20,245.80 to 21,701.94) per 100,000, and the ASPR in 2019 was 20,146.35 per 100,000 (19,407.85 to 20,888.54), with an AAPC of −0.14% (95% CI: −0.15 to −0.12). The age-standardized DALY rate decreased from 751.98 per 100,000 in 1990 (95% CI: 499.20 to 1094.41) to 698.90 per 100,000 in 2019 (466.54 to 1015.31), with an AAPC of −0.25% (95% CI: −0.28% to −0.22%). As shown in Figure 1 and Supplementary Table S1, the global ASPR has slowly declined since 1990, with the most significant decline in ASPR occurring between 2015 and 2019 (APC: −0.52% (−0.56% to −0.48%), *p* < 0.05). Similarly, there was a slight decrease in age-standardized DALY rates for ID in children with different APCs, which declined most significantly between 2017 and 2019 (APC: −1.13% (−1.34% to −0.93%), *p* < 0.05).

**Table 1:** Global prevalence of ID among children in 1990 and 2019 and the AAPC from 1990 to 2019.

Prevalence	1990		2019		1990–2019
	Cases no. (95%UI)	ASR/100,000 (95% CI)	Cases no. (95%UI)	ASR/100,000 (95% CI)	AAPC (%) (95%CI)
Global	371021617.39 (362585126.84 to 379230144.11)	20972.41 (20245.80 to 21701.94)	391491699.23 (382834300.61 to 400499411.06)	20146.35 (19407.85 to 20888.54)	−0.14 (−0.15 to −0.12)
Sex					
Boy	186970624.89 (181355712.01 to 192455285.93)	20536.79 (19520.21 to 21623.95)	199097289.22 (192436644.53 to 205993829.95)	19876.25 (18772.73 to 21033.99)	−0.11 (−0.12 to −0.10)
Girl	184050992.50 (178933064.24 to 188967722.99)	21427.59 (20495.42 to 22361.34)	192394410.01 (187056312.84 to 197567478.57)	20433.52 (19553.32 to 21314.45)	−0.16 (−0.18 to −0.14)
SDI					
High	11630759.09 (10600838.92 to 12606533.17)	6845.61 (5886.15 to 7949.87)	7371084.95 (6492145.97 to 8365813.21)	4671.08 (3765.88 to 5786.17)	−1.30 (−1.35 to −1.25)
High-middle	41285179.29 (39345606.43 to 43401737.4)	13681.96 (12561.77 to 14837.86)	22437432.98 (21042722.77 to 23933637.88)	9271.51 (8331.91 to 10291.10)	−1.34 (−1.37 to −1.31)
Middle	104896699.75 (101000282.37 to 108886457.4)	18005.83 (16949.64 to 19111.67)	74903749.56 (71788498.78 to 77947951.98)	13720.54 (12825.23 to 14632.6)	−0.93 (−0.95 to −0.92)
Low-middle	131071853.43 (126533157.28 to 135237914.75)	28464.13 (26922.54 to 29959.92)	131645173.97 (126737773.98 to 136926308.21)	25497.06 (24067.64 to 27050.28)	−0.38 (−0.39 to −0.36)
Low	81935147.88 (79685554.65 to 84239125.34)	32869.73 (31349.02 to 34380.15)	154901334.64 (150630641.37 to 159216170.24)	32516.96 (30993.53 to 34036.61)	−0.04 (−0.06 to −0.02)
GBD regions					
Western Sub-Saharan Africa	30730276.42 (29391762.94 to 32086254.64)	33841.93 (31364.82 to 36261.61)	73558516.47 (69129405.58 to 77971916.44)	36715.50 (33278.85 to 40025.55)	0.28 (0.26 to 0.30)
South Asia	154505860.98 (149259600.43 to 159314925.74)	34723.8 (32939.56 to 36461.51)	160105820.65 (153978399.23 to 166417752.19)	31523.88 (29637.75 to 33458.24)	−0.32 (−0.34 to −0.31)
Eastern Sub-Saharan Africa	26783289.53 (25836793.43 to 27768762.45)	28726.34 (27148.14 to 30379.36)	47854083.91 (45913937.72 to 49880313.35)	26843.23 (25055.24 to 28692.6)	−0.24 (−0.25 to −0.22)
Central Sub-Saharan Africa	6982519.56 (6224702.74 to 7720404.32)	26029.38 (21307.56 to 30808.8)	15180080.69 (13477412.75 to 16882954.38)	26308.13 (21812.73 to 31108.19)	0.01 (−0.04 to 0.06)
Oceania	603969.06 (537450.29 to 674367.56)	22531.89 (18455.82 to 26856.42)	1111810.99 (962617.26 to 1258941.72)	22370.10 (17445.45 to 27781.41)	−0.03 (−0.07 to 0.01)
Caribbean	2339167.85 (2132360.12 to 2545987.37)	20341.59 (17538.42 to 23485.2)	2488561.19 (2256989.54 to 2724735.35)	21502.95 (18148.53 to 25052.68)	0.21 (0.17 to 0.25)
Central Asia	5909378.56 (5481511.36 to 6333784.05)	23090.65 (20283.31 to 26134.89)	5232945.13 (4,653,920 to 5800774.5)	19244.32 (15835.29 to 22913.75)	−0.63 (−0.67 to −0.59)
Southern Sub-Saharan Africa	3480857.51 (3041278.83 to 3945325.87)	17042.33 (13290.04 to 20981.04)	3605687.95 (3152947.92 to 4109775.01)	15304.75 (12325.12 to 18938.37)	−0.36 (−0.43 to −0.30)
North Africa and Middle East	27374613.92 (25709900.23 to 29186874.75)	18628.01 (16579.13 to 20809.68)	26452064.6 (24566356.7 to 28671263.26)	15187.70 (13391.61 to 17160.04)	−0.71 (−0.75 to −0.66)
Andean Latin America	3267031.63 (2924305.82 to 3683042.76)	21594.63 (17534.67 to 26081.95)	2686720.94 (2298807.32 to 3102904.35)	14834.80 (11554.93 to 18852.94)	−1.30 (−1.34 to −1.25)
Southeast Asia	35886395.92 (33261648.95 to 38777988.56)	20876.28 (18310.71 to 23,622)	20925216.27 (18966205.38 to 22979648.51)	12736.34 (10952.83 to 14777.29)	−1.70 (−1.75 to −1.65)
Tropical Latin America	8811643.93 (7281939.21 to 10611124.08)	16556.42 (11756.46 to 22346.97)	6088968.75 (4840166.53 to 7543103.73)	12536.16 (8376.69 to 17678.4)	−0.95 (−0.97 to −0.94)
Southern Latin America	2167578.37 (1861322.7 to 2523681.77)	14596.41 (11178.44 to 18709.95)	1456907.44 (1113640.66 to 1833745.98)	10144.72 (6595.32 to 14813.74)	−1.25 (−1.31 to −1.19)
Central Europe	3737058.78 (3348518.12 to 4151207.76)	13531.36 (11283.52 to 16029.86)	1664081.59 (1456146.38 to 1911562.33)	9797.95 (7831.49 to 12275.83)	−1.10 (−1.16 to −1.04)
Central Latin America	7918364.25 (7451470.98 to 8451980.79)	12269.9 (11084.05 to 13618.52)	5804863.12 (5379536.02 to 6271630.9)	9114.43 (8124.54 to 10266.49)	−1.01 (−1.03 to −0.99)
Australasia	436887.97 (314627.13 to 599719.08)	9769.42 (5933.53 to 15445.22)	378781.56 (255618.87 to 550578.67)	7152.01 (4056.56 to 12186.55)	−1.07 (−1.09 to −1.05)
High-income Asia Pacific	3427967.90 (2797716.68 to 4168468.3)	10227.36 (7136.79 to 14450.51)	1426249.47 (1050589.26 to 1879055.29)	6350.17 (3884.4 to 10061.75)	−1.63 (−1.64 to −1.61)
Eastern Europe	3651014.64 (3044970.86 to 4315915.64)	7212.68 (5325.01 to 9604.88)	1,799,697 (1330382.64 to 2402253.96)	4951.41 (3124.79 to 7738.71)	−1.30 (−1.39 to −1.22)
Western Europe	4447491.03 (3921527.96 to 5044839.61)	6502.45 (5220.08 to 8021.81)	2784386.85 (2296580.05 to 3337399.75)	4219.94 (3198.71 to 5551.05)	−1.48 (−1.51 to −1.46)
East Asia	36157198.73 (33082772.51 to 39382246.06)	10732.14 (9162.45 to 12413.07)	8555501.51 (7185285.86 to 10167906.68)	3629.73 (2723.62 to 4711.96)	−3.67 (−3.78 to −3.57)
High-income North America	2403050.86 (1934127.33 to 2891510.42)	3911.74 (2686.7 to 5487.18)	2330753.13 (1678762.64 to 3118717.59)	3618.47 (2053.33 to 5826.49)	−0.27 (−0.35 to −0.19)

**Table 2:** Global DALYs of ID among children in 1990 and 2019 and AAPC from 1990 to 2019.

DALY	1990		2019		1990–2019
	Cases no. (95%UI)	ASR/100,000 (95% CI)	Cases no. (95%UI)	ASR/100,000 (95% CI)	AAPC (%) (95%CI)
Global	13265334.81 (8868971.7 to 19146397.21)	751.98 (499.20 to 1094.41)	13620230.64 (9174233.54 to 19794594.83)	698.90 (466.54 to 1015.31)	−0.25 (−0.28 to −0.22)
Sex					
Boy	6586677.11 (4382196.30 to 9587842.03)	725.74 (480.82 to 1056.29)	6847447.67 (4551907.88 to 10070226.66)	681.77 (448.37 to 1002.33)	−0.21 (−0.25 to −0.18)
Girl	6678657.69 (4487320.53 to 9597565.14)	779.53 (518.29 to 1125.16)	6772782.97 (4536543.65 to 9810175.15)	717.15 (476.56 to 1042.48)	−0.29 (−0.32 to −0.26)
SDI					
High SDI	269104.95 (170,046 to 400870.35)	157.31 (96.94 to 241.84)	149955.44 (93839.06 to 231790.76)	93.68 (55.55 to 149.37)	−1.77 (−1.8 to −1.73)
High-middle SDI	1271719.00 (835147.15 to 1882272.61)	421.01 (268.58 to 619.83)	618841.96 (402987.94 to 911054.32)	254.57 (162.91 to 385.17)	−1.73 (−1.76 to −1.69)
Middle SDI	3514211.28 (2316672.84 to 5141554.65)	603.91 (396.74 to 878.07)	2336857.73 (1555153.16 to 3391386.24)	425.65 (282.68 to 624.46)	−1.20 (−1.22 to −1.17)
Low-middle SDI	4965142.94 (3304139.23 to 7163848.93)	1083.47 (713.64 to 1574.43)	4611592.66 (3079567.4 to 6738220.99)	887.81 (585.77 to 1297.56)	−0.68 (−0.69 to −0.67)
Low SDI	3238741.22 (2185104.53 to 4708582.34)	1310.76 (875.23 to 1898.93)	5895716.72 (3944808.94 to 8616020.1)	1240.83 (819.96 to 1813.62)	−0.19 (−0.21 to −0.17)
GBD regions					
Western Sub-Saharan Africa	1222710.22 (813347.31 to 1762107.47)	1354.21 (881.91 to 1991.02)	2847854.97 (1885088.17 to 4113576.07)	1425.30 (921.99 to 2097.08)	0.18 (0.16 to 0.19)
South Asia	6180310.54 (4176161.21 to 8836557.46)	1394.55 (926.82 to 1992.55)	6046176.40 (4004177.84 to 8790538.78)	1180.06 (775.65 to 1732.25)	−0.57 (−0.58 to −0.56)
Eastern Sub-Saharan Africa	1015334.87 (673858.07 to 1469945.44)	1097.35 (723.36 to 1587.21)	1653748.53 (1104166.98 to 2426650.9)	931.01 (613.91 to 1366.16)	−0.57 (−0.61 to −0.53)
Central Sub-Saharan Africa	240571.03 (154332.58 to 358341.65)	910.91 (557.89 to 1403.29)	496116.51 (315481.14 to 735381.39)	863.04 (516.59 to 1321.32)	−0.21 (−0.30 to −0.12)
Oceania	20876.09 (13282.6 to 30499.01)	784.05 (464.61 to 1210.33)	37276.48 (23079.6 to 55039.4)	756.95 (441.32 to 1199.61)	−0.13 (−0.16 to −0.09)
Caribbean	69445.94 (44316.1 to 103816.4)	607.02 (369.47 to 925.28)	75295.50 (48559.93 to 113864.02)	648.64 (391.18 to 1006.31)	0.24 (0.19 to 0.28)
Central Asia	192083.31 (125716.29 to 280014.93)	756.68 (475.62 to 1149.63)	154800.50 (98709.2 to 227226.4)	570.05 (340.27 to 877.65)	−0.98 (−1.01 to −0.94)
Southern Sub-Saharan Africa	104867.32 (66195.05 to 156323.47)	514.16 (301.1 to 813.69)	106635.70 (67522.64 to 159646.54)	451.45 (264.25 to 704.9)	−0.44 (−0.53 to −0.34)
North Africa and Middle East	853493.49 (563829.98 to 1261457.22)	583.27 (376.3 to 866.5)	786372.80 (515429.96 to 1178235.7)	450.83 (283.09 to 684.98)	−0.88 (−0.93 to −0.84)
Andean Latin America	110575.19 (71283.56 to 165935.88)	733.3 (445.26 to 1141.98)	70861.47 (44016.3 to 110157.65)	391.54 (224.06 to 640.84)	−2.15 (−2.22 to −2.07)
Tropical Latin America	301659.79 (184383.07 to 458878.67)	564.04 (301.54 to 939.29)	182219.01 (110308.6 to 283954.34)	372.9 (193.08 to 648.09)	−1.41 (−1.43 to −1.39)
Southeast Asia	1135042.15 (744924.01 to 1714570.04)	659.59 (418.95 to 1006.28)	569630.48 (358447.8 to 864036.43)	343.49 (212.38 to 529.35)	−2.23 (−2.30 to −2.16)
Central Europe	104840.17 (66023.39 to 156111.53)	374.31 (225.5 to 580.94)	39486.66 (24252.71 to 60494.09)	229.62 (133.09 to 368.52)	−1.66 (−1.73 to −1.59)
Central Latin America	225856.01 (146134.5 to 332869.59)	351.26 (225.77 to 523.15)	143777.06 (93522.31 to 210590.62)	224.15 (142.47 to 336.01)	−1.53 (−1.56 to −1.51)
Southern Latin America	57386.1 (34,957 to 90704.32)	386.27 (212.73 to 643.7)	31920.77 (18627.47 to 51889.33)	220.66 (109.36 to 413.86)	−1.93 (−2.03 to −1.83)
High-income Asia Pacific	92962.55 (55825.73 to 143062.07)	271.06 (148.56 to 454.08)	31930.17 (18161.51 to 52886.74)	139.62 (65.88 to 258.26)	−2.26 (−2.28 to −2.24)
Australasia	8075.67 (4447.01 to 14469.79)	179.97 (81.63 to 371.61)	6195.33 (3133.51 to 11129.22)	116.21 (47.28 to 244.48)	−1.50 (−1.52 to −1.47)
Eastern Europe	82534.81 (51439.66 to 128088.5)	161.74 (88.67 to 274.67)	35945.10 (20388.75 to 59327.65)	97.8 (47.08 to 183.49)	−1.74 (−1.83 to −1.65)
East Asia	1111198.18 (709307.92 to 1620997.66)	330.69 (198.94 to 504.97)	206133.79 (127438.05 to 317068.12)	87.97 (47.57 to 145.28)	−4.49 (−4.57 to −4.40)
Western Europe	89831.76 (55788.6 to 137744.3)	130.02 (77.32 to 203.8)	52145.66 (31613.08 to 81175.06)	77.99 (44.32 to 129.03)	−1.75 (−1.77 to −1.73)
High-income North America	45679.64 (26652.77 to 71822.76)	74.44 (38.9 to 129.2)	45707.74 (24972.58 to 78926.71)	69.36 (30.06 to 133.63)	−0.24 (−0.29 to −0.18)

### Age and sex patterns

**Figure 2:**
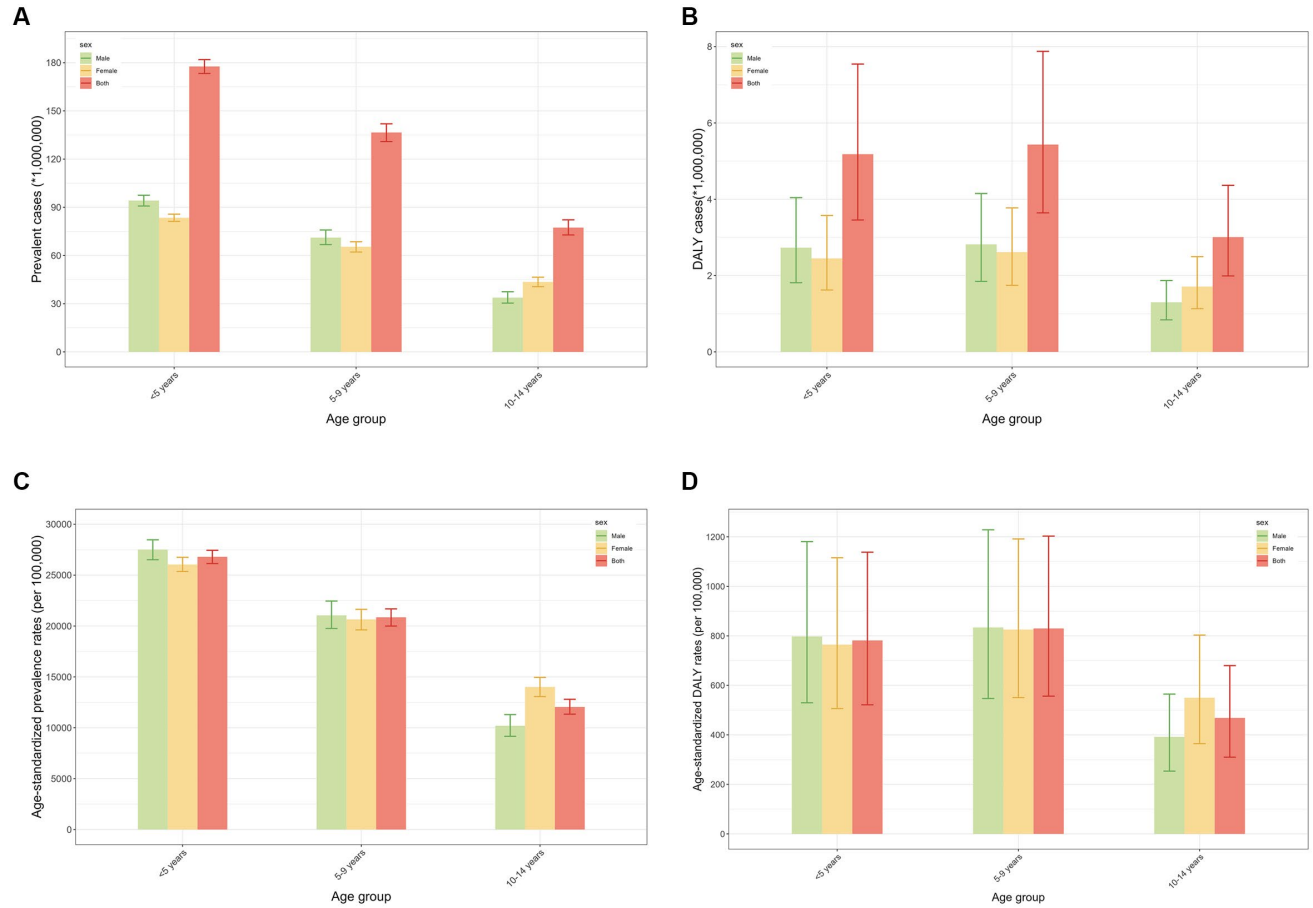
Prevalence and DALY of global children with ID in different age groups in 2019. **(A)** Prevalent cases in different age groups in 2019; **(B)** Prevalence rates in different age groups in 2019; **(C)** DALY cases in different age groups in 2019; **(D)** DALY rates in different age groups in 2019. DALY, disability-adjusted life year; ID, iron deficiency.

Globally, trends in the number of prevalent cases, ASPR, DALYs, and age-standardized DALY rate by sex and age group from 1990 to 2019 are shown in Supplementary Tables S2–S5, Supplementary Figures S1–S3, and Figure 2. With respect to gender, the prevalence of ID in children was more pronounced in girls than in boys, as was the case for DALY. The ASPR for ID has declined over the past three decades in both boys and girls, with an AAPC of −0.11% [95% CI: −0.12% to −0.10%; from 20,536.79 (95% CI: 19,520.21 to 21,623.95) per 100,000 in 1990 to 19,876.25 (18,772.73 to 21,033.99) per 100,000 in 2019] in boys, and − 0.16% [−0.18% to −0.14%; from 21,427.59 (20,495.42 to 22,361.34) per 100,000 to 20,433.52 (19,553.32 to 21,314.45) per 100,000] in girls. Similarly, the age-standardized DALY rate declined. The AAPC for males is −0.21% [95% CI: −0.25% to −0.18%; decreasing from 725.74 (95% CI: 480.82 to 1056.29) per 100,000 in 1990 to 681.77 (448.37 to 1002.33) per 100,000 in 2019], and for females is −0.29% [−0.32% to −0.26%; from 779.53 (518.29 to 1125.16) per 100,000 in 1990 to 717.15 (476.56 to 1042.48) per 100,000 in 2019].

In terms of age, the highest prevalence in the last 30 years was in the age group of less than 5 years. The results of the Joinpoint regression analyses showed a decreasing trend in prevalence between 1990 and 2019 in the <5 years age group [AAPC: −0.29% (95% CI: −0.32% to −0.25%), *p* < 0.05], with the most pronounced decrease between 2006 and 2019 [APC: −0.76% (−1.00% to −0.52%), *p* < 0.05]. DALY rates for ID declined in all three age groups during this period, with the greatest decline in those younger than 5 years of age [AAPC: −0.54% (95% CI: −0.58% to −0.50%)], and the most pronounced decline from 2001 to 2010 [APC: −0.94% (−0.98% to −0.91%), *p* < 0.05]. In 2019, the DALY rate was highest in the 5–9 years age group [829.78 (95% CI: 556.53 to 1203.08) per 100,000].

### Association with the socio-demographic index

**Figure 3:**
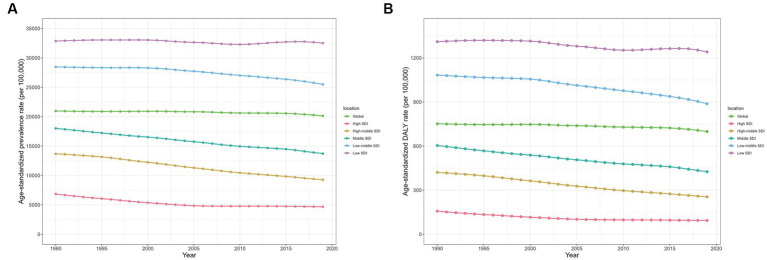
Prevalence and DALY rates of children with ID in five SDI regions from1990 to 2019. **(A)** Prevalence; **(B)** DALY rates. DALY, disability-adjusted life year; ID, iron deficiency.

ID burden among children in different SDI regions is presented in Tables 1, 2 and Figure 3. From 1990 to 2019, the prevalence rates in all five SDI regions showed a decreasing trend, with the largest decrease occurring among those with high-middle SDI [from 13,681.96 (95% CI: 12,561.77 to 14,837.86) per 100,000 in 1990 to 9271.51 (8331.91 to 10,291.10) per 100,000 in 2019; AAPC: −1.34% (95%CI: −1.37% to −1.31%)], followed by high SDI areas. The highest prevalence rates in 2019 were found in the low-SDI quintile countries [32,516.96 (95%CI: 30,993.53 to 34,036.61) per 100,000], and the high-SDI quintile countries [4671.08 (3765.88 to 5786.17) per 100,000] had the lowest estimates.

**Figure 4:**
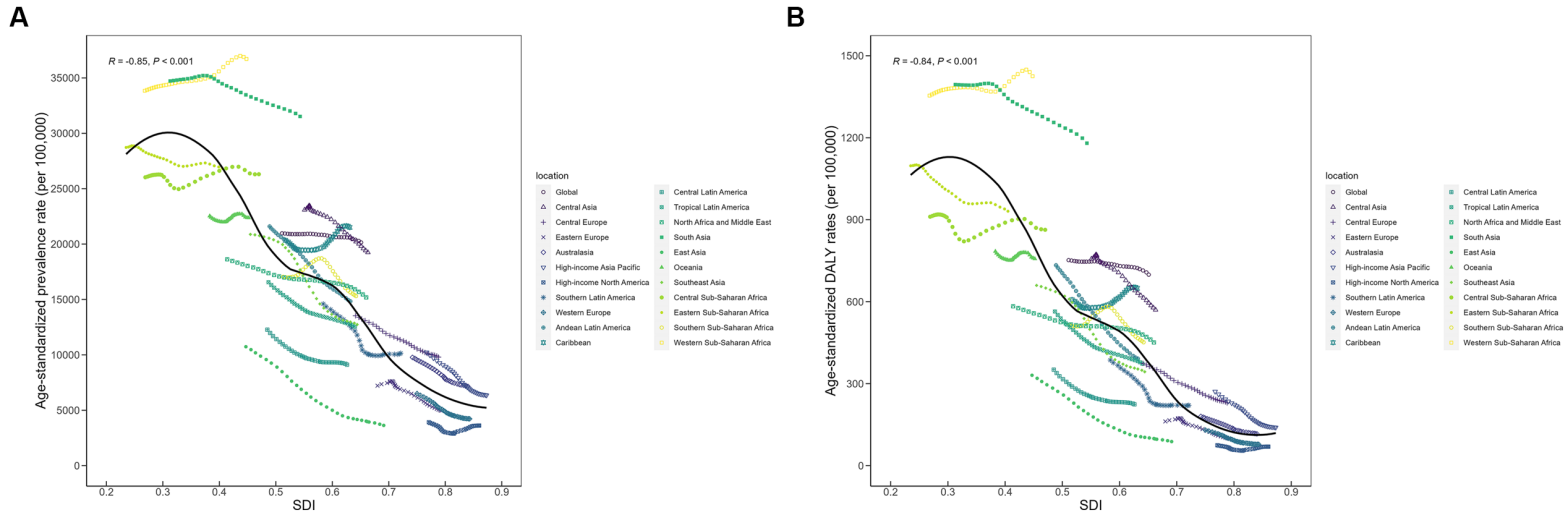
Prevalence and DALY rates of children with ID across 21 GBD regions by socio-demographic index. **(A)** Prevalence; **(B)** DALY rates. DALY, disability-adjusted life year; ID, iron deficiency.

DALY rates for ID have also declined in all five SDI regions over the past 30 years, with the most pronounced decline in the high SDI region [from 157.31 (95% CI: 96.94 to 241.84) per 100,000 in 1990 to 93.68 (55.55 to 149.37) per 100,000 in 2019; AAPC: −1.77% (95%CI: −1.80% to −1.73%)], followed by the high-middle SDI region. The highest DALY rate in 2019 was found in the low SDI quintile countries [1240.83 (95% CI: 819.96 to 1813.62) per 100,000] and the lowest in the high SDI quintile countries [93.68 (55.55 to 149.37) per 100,000]. The variations in ASPR and DALY rates of ID among children across SDI by 21 GBD regions are shown in Figure 4. Overall, there was a negative correlation between ASPR and SDI across global regions from 1990 to 2019, as well as age-standardized DALY rate.

### Regional level

At the regional level, from 1990 to 2019, East Asia [from 10,732.14 (95% CI: 9162.45 to 12,413.07) per 100,000 in 1990 to 3629.73 (2723.62 to 4711.96) per 100,000 in 2019; AAPC: −3.67% (95% CI: −3.78% to −3.57%)], Southeast Asia [from 20,876.28 (18,310.71 to 23,622.00) per 100,000 in 1990 to 12,736.34 (10,952.83 to 14,777.29) per 100,000 in 2019; AAPC: −1.70% (−1.75% to −1.65%)], and High-income Asia Pacific [from 10,227.36 (7136.79 to 14,450.51) per 100,000 in 1990 to 6350.17 (3884.40 to 10,061.75) per 100,000 in 2019; AAPC: −1.63% (−1.64% to −1.61%)] had the largest decrease in the prevalence of ID among children. In contrast, ASPR increased in the Caribbean [AAPC: 0.21% (95% CI: 0.17 to 0.25%)] and Western Sub-Saharan Africa [0.28% (0.26 to 0.30%)]. Apparently, in 2019, Western Sub-Saharan Africa [36,715.50 (95% CI: 33,278.85 to 40,025.55) per 100,000], South Asia [31,523.88 (29,637.75 to 33,458.24) per 100,000], and Eastern Sub-Saharan Africa [31,523.88 (29,637.75 to 33,458.24) per 100,000] showed the highest ASPRs, whereas the regions with the lowest ASPRs were High-income North America [3618.47 (2053.33 to 5826.49) per 100,000], East Asia [3629.73 (2723.62 to 4711.96) per 100,000] and Western Europe [4219.94 (3198.71 to 5551.05) per 100,000].

From 1990 to 2019, age-standardized DALY rates tended to decline in the vast majority of geographic regions, especially in East Asia [from 330.69 (95% CI: 198.94 to 504.97) per 100,000 in 1990 to 87.97 (47.57 to 145.28) per 100,000 in 2019; AAPC: −4.49% (95% CI: −4.57% to −4.40%)], Southeast Asia [from 659.59 (418.95 to 1006.28) per 100,000 in 1990 to 343.49 (212.38 to 529.35) per 100,000 in 2019; AAPC: −2.23% (−2.30% to −2.16%)] and High-income Asia Pacific [from 271.06 (148.56 to 454.08) per 100,000 in 1990 to 139.62 (65.88 to 258.26) per 100,000 in 2019; AAPC: −2.26% (−2.28% to −2.24%)]. Notably, in 2019, Western Sub-Saharan Africa [1425.30 (95% CI: 921.99 to 2097.08) per 100,000], South Asia [1180.06 (775.65 to 1732.25) per 100,000], and Eastern Sub-Saharan Africa [931.01 (613.91 to 1366.16) per 100,000] were the regions with the highest DALYs due to ID among children.

### National level

**Figure 5:**
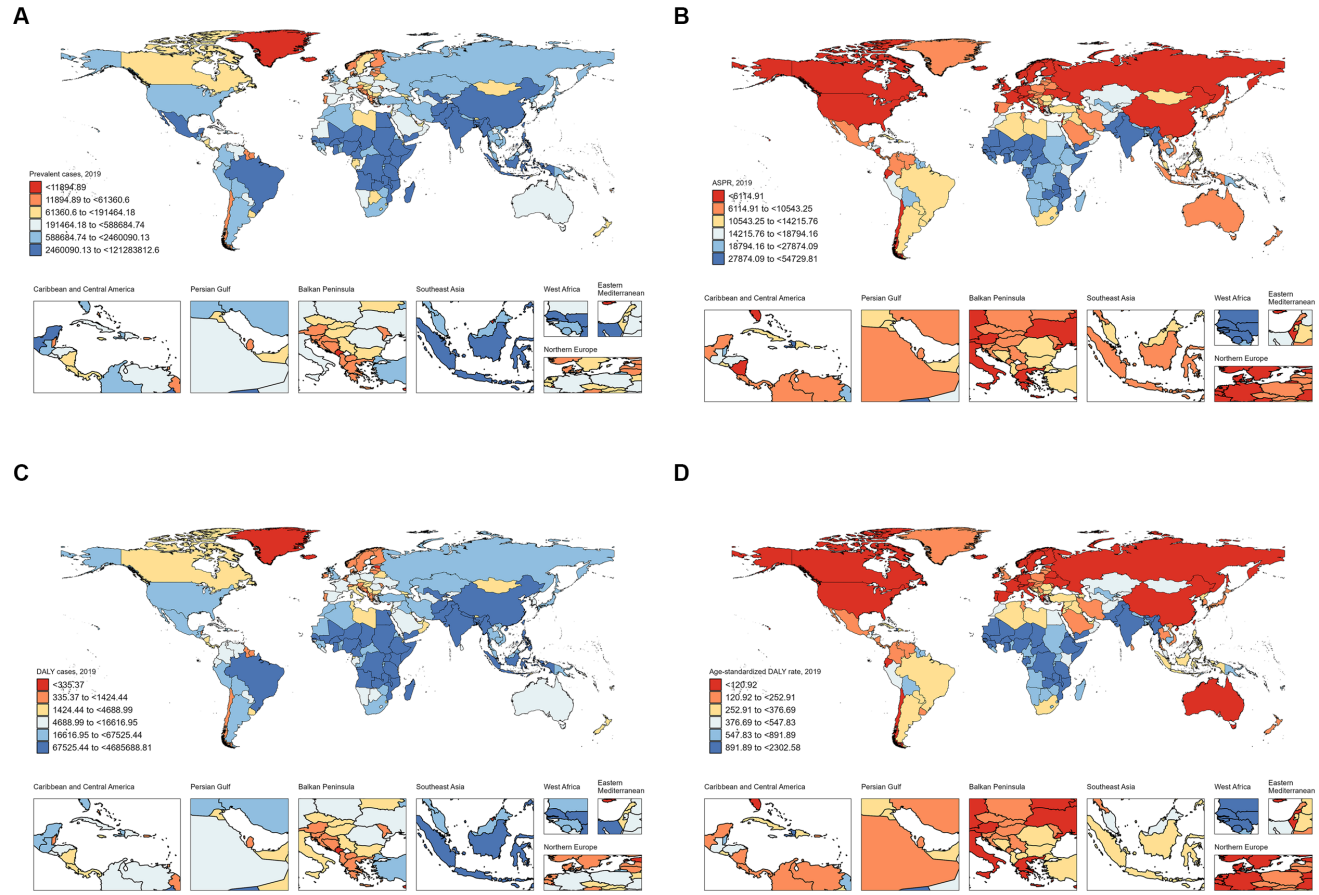
Prevalence and DALY of children with ID in different countries and territories in 2019. **(A)** Prevalent cases; **(B)** Prevalence; **(C)** DALY cases; **(D)** DALY rates. DALY, disability-adjusted life year; ID, iron deficiency.

Details of ID burden among children in various countries and territories are presented in Supplementary Tables S6, S7 and Figure 5. Nationally, the prevalence of ID in children declined in most countries, with the most pronounced decline in Ecuador [from 18,161.05 (95% CI: 12,274.35 to 25,036.33) per 100,000 in 1990 to 5685.00 (3472.33 to 8622.14) per 100,000 in 2019; EAPC: −4.52 (95%CI: −4.71 to −4.32)], followed by China [from 10,604.68 (9006.75 to 12,325.94) per 100,000 in 1990 to 3415.32 (2478.93 to 4529.54) per 100,000 in 2019; EAPC: −4.26 (−4.51 to −4.01)] and Chile [from 3864.85 (1778.45 to 7758.28) per 100,000 in 1990 to 1543.07 (600.89 to 3446.53) per 100,000 in 2019; EAPC: −3.05 (−3.31 to −2.79)]. Conversely, prevalence rates increased in a few countries, with Burkina Faso [from 29,783.32 (95% CI: 23,608.87 to 36,051.48) per 100,000 in 1990 to 42,571.67 (34,401.56 to 51,755.86) per 100,000 in 2019; EAPC: 1.43 (95% CI: 1.31 to 1.55)], Angola [from 15,982.47 (11,121.66 to 21,501.39) per 100,000 in 1990 to 22,108.10 (15,928.42 to 29,531.69) per 100,000 in 2019; EAPC: 1.55 (1.40 to 1.69)], and Yemen [from 24,791.91 (20,746.81 to 29,176.45) per 100,000 in 1990 to 33,594.07 (28,665.26 to 38,997.44) per 100,000 in 2019; EAPC: 1.36 (1.22 to 1.50)] having the greatest increase. Evidently, in 2019, Bhutan [54,729.81 (95% CI: 46,877.72 to 62,083.13) per 100,000], Mali [47,477.39 (40,291 to 54,123.33) per 100,000] and Gambia [45,473.78 (38,237.83 to 52,214.25) per 100,000] had the highest ASPR, while the countries with the lowest ASPR were Chile [1543.07 (600.89 to 3446.53) per 100,000] and Canada [2023.79 (833.01 to 4157.10) per 100,000].

From 1990 to 2019, a decreasing trend in the age-standardized DALY rate was observed in most of countries, with Ecuador [from 524.27 (95% CI: 273.37 to 899.41) per 100,000 in 1990 to 120.31 (57.91 to 224.17) per 100,000 in 2019; EAPC: −5.61 (95%CI: −5.83 to −5.38)] having the largest decline. On the contrary, Burkina Faso [from 1150.14 (95% CI: 635.05 to 1864.62) per 100,000 in 1990 to 1801.44 (1055.54 to 2822.90) per 100,000 in 2019; EAPC. 1.78 (95% CI: 1.66 to 1.90)] experienced the largest increase during this period. Notably, in 2019, Bhutan [2302.58 (95% CI: 1405.02 to 3478.33) per 100,000], Mali [2151.80 (1316.27 to 3221.81) per 100,000] and Burkina Faso [1801.44 (1055.54 to 2822.9) per 100,000] were the countries with the highest DALY rates.

## Discussion

ID is the most common micronutrient deficiency worldwide. ID among children triggers a number of health consequences, such as significantly affecting growth and development, impairing the cognitive development of the brain, diminishing immune function, and affecting the metabolism of other nutrients (23–26). There has been some research showing concern about ID in children (27–29). This study provided a new and comprehensive assessment of the disease burden of ID in children, which will facilitate appropriate preventive measures for childhood ID by regional governments. Strengthening the prevention and treatment of childhood ID has important public health significance for reducing the global burden of disease.

Based on our findings, ID prevalence varied by age and gender, suggesting that age and gender can be used to promote targeted ID screening among children. Since 1990, thanks to worldwide efforts, the global prevalence and DALY rates of ID in children have continued to decline slowly. However, between 1990 and 2019, those younger than five years old were the only age group with a significant decrease in ID prevalence. Despite this, the prevalence is still remarkably higher in children younger than five years old, suggesting that this group is considered to be at high risk for the onset of ID. This may be closely related to increased iron requirements due to rapid growth and development. Given that early childhood is a crucial period for intellectual and psychomotor development, measures to prevent ID should continue to be supported, encouraged and implemented.

Gender differences in ID burden varied across age groups. As a whole, ID prevalence was higher in girls than in boys among children younger than 15 years old. During this period, the prevalence and DALY rates were higher in girls than in boys for children aged 10 to 14 years. Whereas the other two age groups appeared to have a higher prevalence in boys than in girls. In other words, our results indicated that boys under 10 years of age endure a heavier burden of ID than girls worldwide. Consistent with our findings, several studies have reported lower iron stores in male infants and children, which may put them at greater risk for IDA (30, 31). Conversely, other studies have demonstrated girls to have higher rates of anemia than boys (32). That is, public health messages have consistently emphasized that girls are a high-risk group, so mothers of young girls may prioritize their dietary iron needs over those of boys, resulting in higher iron sufficiency rates for girls and greater deficiency in boys (33).

Socio-economic factors also play an important role in childhood ID, with East Asia experiencing the greatest decline in prevalence and DALY rates between 1990 and 2019. Regionally, from 1990 to 2019, age-standardized prevalence DALY rates tended to decline in the vast majority of geographic regions. However, we found an increase in ID prevalence and DALY rates among children in western sub-Saharan Africa, and it is critical to determine the underlying causes of the increased estimates. Western Sub-Saharan Africa, South Asia, and Eastern Sub-Saharan Africa continue to have high prevalence and DALY rates, which may be mainly due to insufficient dietary supply of bioavailable iron to meet children’s needs. The causes of ID vary widely at different stages of life, as well as between gender and socio-economic circumstances (34). The scientific feeding of parents, the level of parental knowledge, and the number of children in the family are also associated with the occurrence of children with ID. Notably, micronutrient deficiencies (such as vitamin A, vitamin C, etc.) lead to poor iron absorption, which is particularly evident in children aged 0–6 years. Anemia is one of the many consequences of ID (35). IDA affects cognitive functioning in children, delays motor development, impairs physical functioning and quality of life, and poses a major global health problem and challenge for developing countries (35).

At the national level, India (121,283,813), Nigeria (36,200,362) and Pakistan (27,331,933) had the highest number of prevalent cases in 2019. Iron deficiency anemia (IDA) prevalence is 49.5% in 6–23-month-old and 39.9% in 24–58-month-old children, which has resulted in huge intangible costs and production losses for Indian society (36). The status of literacy and wealth of parents may be an important factor (37). In this regard, educational interventions by school teachers for children or adolescents may be effective in raising their awareness and attitudes towards diseases and their prevention (38). Similarly, Nigeria (39) and Pakistan (40) face the same social problem – ID in children. Bhutan, Mali, and Gambia shouldered the highest ASPR. The prevalence of anemia among preschool children in Bhutan was as high as 42% according to the Bhutan National Nutrition Survey 2015 (41). Notably, we found a more than 35 times difference in the prevalence of ID in children between different countries and territories, ranging from 1,543 to 54,730 per 100,000. It is noteworthy that a few countries in the lower SDI quintile experienced an increase in prevalence during the study period, including Burkina Faso, Angola, and Yemen. More research is needed to understand the reasons for the increase in ID prevalence in these countries. ID burden among children from countries in the lower SDI quintiles requires urgent attention. The country with the largest decrease in prevalence was Ecuador, followed by China and Chile.

There were 7.79 million children with ID in China in 2019, which had decreased by 77.4% since 1990. We found lower age-standardized prevalence and DALY rates in China compared with most of the countries in this study. In 2019, the ASPR for childhood ID in China was 3415.32 per 100,000, which was significantly lower than the estimates for Bhutan (54,729.81 per 100,000) or Mali (47,477.39 per 100,000), but was slightly higher than Italy (3311.53 per 100,000) or Monaco (3270.25 per 100,000). As for the DALY rate of childhood ID, the value for China was 82.6 per 100,000 in 2019, far below worldwide standards (698.9 per 100,000). Overall, the prevalence and DALY rates of childhood ID in China showed a decreasing trend from 1990 to 2019. During this period, the EAPCs of ASPR and DALY rate in China were − 4.26 (95% CI, −4.51 to −4.01) and − 5.14 (−5.43 to −4.84), respectively. Evidently, the improvement in ID burden among Chinese children was much greater than the global level.

ID is disabling. In 2017, the GBD Study reported that dietary ID remains the fourth and twelfth leading cause of years lived with disability in females and males, respectively (42). Age-standardized DALY rates showed a decreasing trend in most regions, especially in East Asia, Southeast Asia, and High-income Asia Pacific, but countries with lower SDI still had the highest DALY rates in 2019. It is critical to promote effective inter-country interventions and collaboration to improve healthcare for children with ID in countries with low SDI.

Countries and regions with a high ID prevalence among children must take effective preventive measures against ID. First of all, the publicity and prevention of ID should be further improved. In accordance with the relevant regulations, children should be provided with regular health screening and nutritional assessments, and education on nutritional knowledge should be strengthened, so as to ensure that children can have reasonable nutrition and balanced diets, and that bad habits such as picky eating can be positively corrected. In addition, children have rapid growth and development, and the amount of iron contained in food usually fails to meet their iron needs. Oral iron is the first line of treatment for ID in children, and iron supplementation treatment can significantly improve children’s lagging growth and development (43).

### Limitations

A strength of the study is that we have provided up-to-date and comprehensive estimates of levels and trends associated with ID among children at the global, regional, and national levels. Nevertheless, there are some limitations of this study. First, we assessed ID burden among children based on age, sex, and SDI, but did not assess its other risk factors. In addition, as the data were aggregated across multiple sites globally, there may be limitations of underdiagnosis and underreporting, which may lead to an underestimation of our results and needs to be approached with caution when interpreting the results. Finally, our study was limited by the variable quality of the GBD data and missing data, and we were unable to analyse incidence rates in this study due to the unavailability of data on ID incidence in the GBD database.

### Areas for further research

To balance the limitations of GBD study, more international cooperation should be encouraged, including annual searches for available data with national collaborators. The quality of the data used in this study relies on the quality control of the original GBD data collection process, and bias is still inevitable. It is suggested that the findings of this study be further validated with the help of large cohort studies. Moreover, the registration of global epidemiological data should be strengthened. Furthermore, the potential reasons for increased prevalence and DALY rates in a few countries are also worthy of attention. Besides, the incidence of childhood ID needs to be further studied, and Bayesian forecasting model is available for future trends in the burden of disease.

## Conclusions and recommendations

Despite the global trend of decreasing prevalence and DALY rates of ID among children between 1990 and 2019, childhood ID still remains an important public health problem in the future due to the rising number of prevalent cases and DALYs, especially in countries with low SDI. Iron fortification remains an essential aspect of childhood health. There is an urgent need for screening and prevention and control of ID for children in economically less developed areas. Children younger than 5 years old are an important population that requires targeted measures to reduce the ID burden. It is imperative to enhance parental awareness of ID and feeding knowledge. Iron-rich foods are recommended to increase the amount of iron available to children through proper dietary intake. In addition, increased food diversity and improved iron bioavailability are also advisable. Moreover, bridging the gap between high-income and low-income countries and carefully defining strategies to reach target populations may facilitate the reduction of ID rates.

## Data availability statement

The original contributions presented in the study are included in the article/Supplementary material, further inquiries can be directed to the corresponding authors.

## Author contributions

DL: Conceptualization, Writing – original draft. CM: Data curation, Writing – original draft. YL: Formal analysis, Writing – review & editing. TZ: Investigation, Writing – review & editing. YX: Project administration, Writing – review & editing. YZ: Project administration, Writing – review & editing.

## Footnotes

^1^ http://ghdx.healthdata.org/

## Supplementary material

Supplementary Figure S1 Joinpoint regression analysis of global ID prevalence and DALY rates in children younger than 5 years old from 1990 to 2019 **(A)** Prevalence; **(B)** DALY rates. DALY, disability-adjusted life year; ID, iron deficiency.

Supplementary Figure S2 Joinpoint regression analysis of global ID prevalence and DALY rates in children aged 5-9 years from 1990 to 2019 **(A)** Prevalence; **(B)** DALY rates. DALY, disability-adjusted life year; ID, iron deficiency.

Supplementary Figure S3 Joinpoint regression analysis of global ID prevalence and DALY rates in children aged 10-14 years from 1990 to 2019 **(A)** Prevalence; **(B)** DALY rates. DALY, disability-adjusted life year; ID, iron deficiency.
