# Supplementary figures and images for: Evolving trends and burden of iron deficiency among children, 1990–2019: a systematic analysis for the global burden of disease study 2019
Source: Front Nutr. 2023 Dec 7;10:1275291. doi: 10.3389/fnut.2023.1275291 (PMC10734639; doi:10.3389/fnut.2023.1275291)

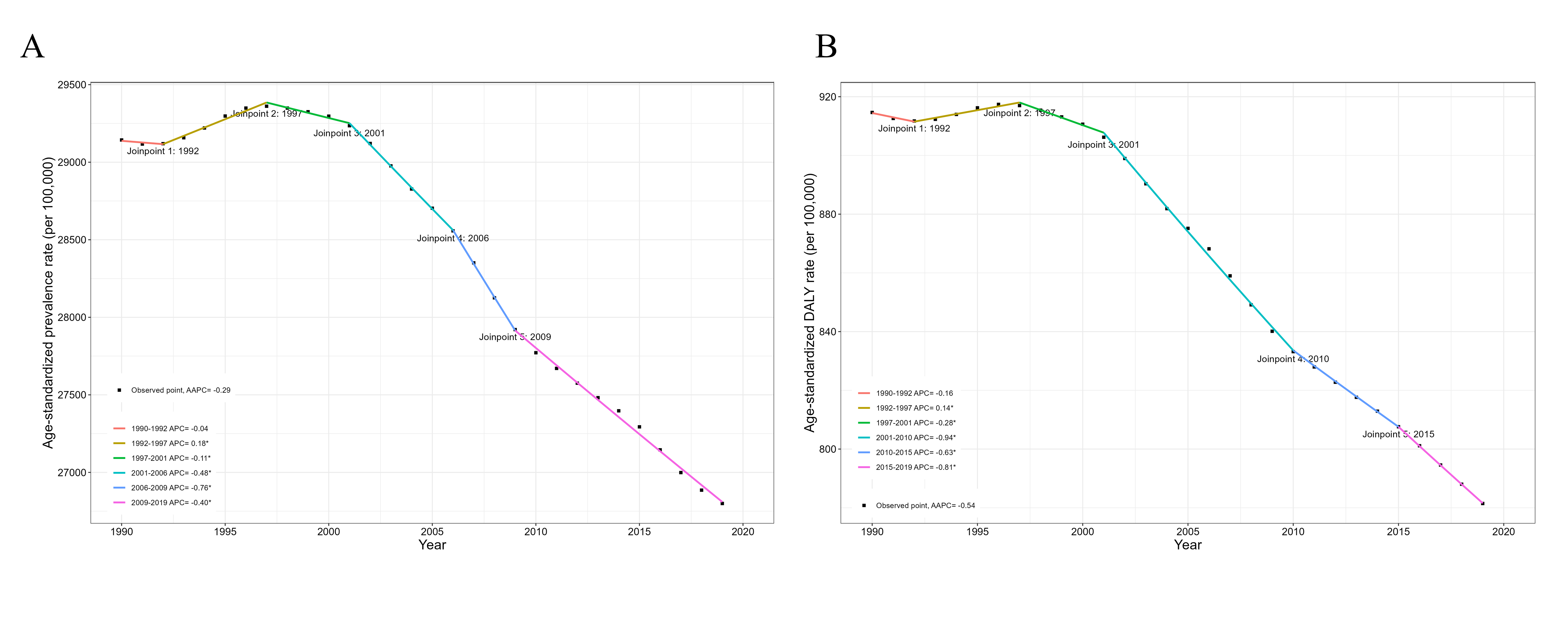

Supplement: Supplementary Figure S1 — Joinpoint regression analysis of global ID prevalence and DALY rates in children younger than 5 years old from 1990 to 2019 (A) Prevalence; (B) DALY rates. DALY, disability-adjusted life year; ID, iron deficiency. [file Image_1.TIF]

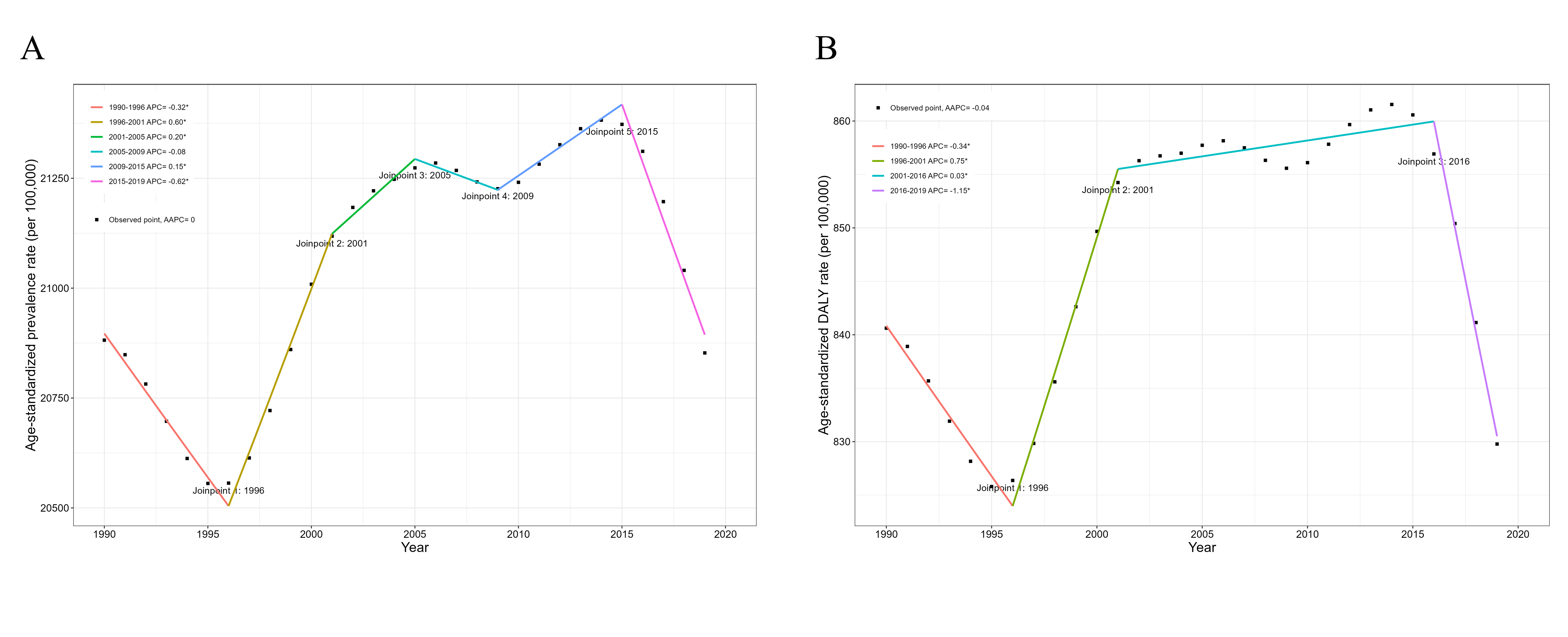

Supplement: Supplementary Figure S2 — Joinpoint regression analysis of global ID prevalence and DALY rates in children aged 5-9 years from 1990 to 2019 (A) Prevalence; (B) DALY rates. DALY, disability-adjusted life year; ID, iron deficiency. [file Image_2.TIF]

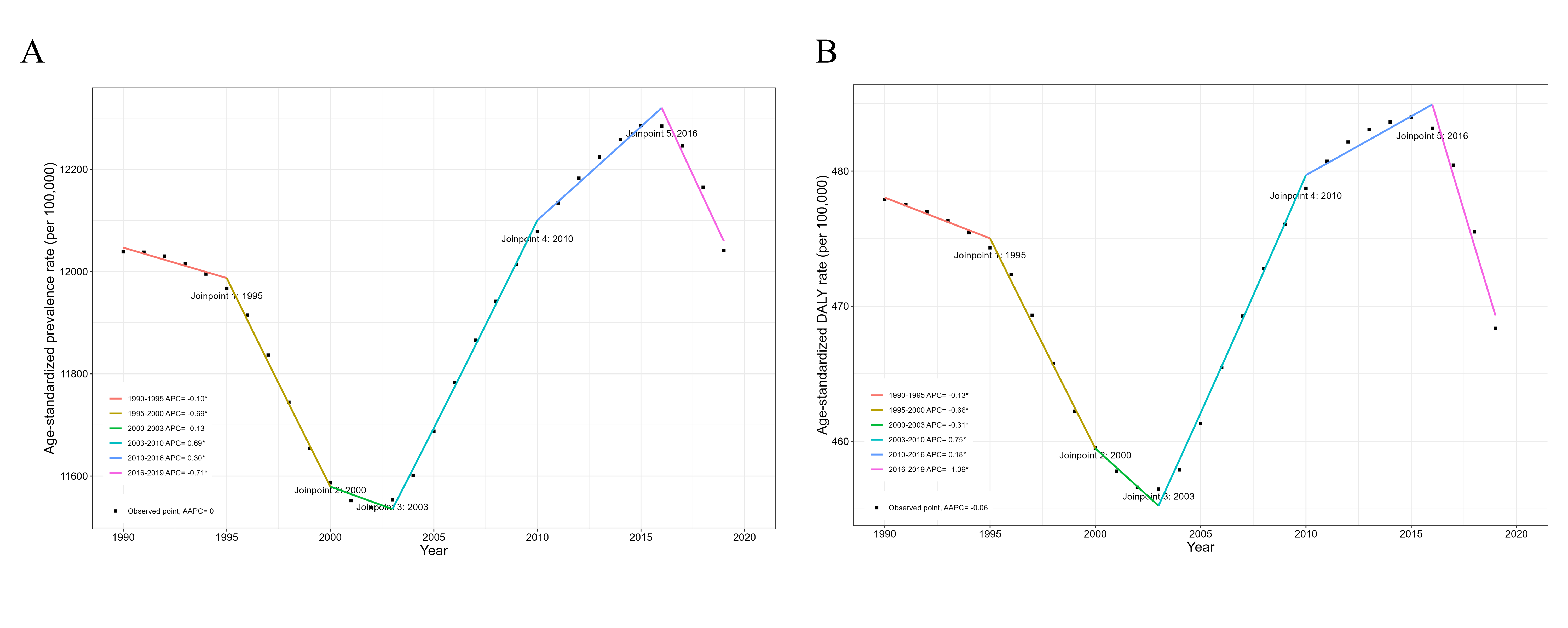

Supplement: Supplementary Figure S3 — Joinpoint regression analysis of global ID prevalence and DALY rates in children aged 10-14 years from 1990 to 2019 (A) Prevalence; (B) DALY rates. DALY, disability-adjusted life year; ID, iron deficiency. [file Image_3.TIF]
