# Supplementary material for: Evolving trends and burden of iron deficiency among children, 1990–2019: a systematic analysis for the global burden of disease study 2019
Source: Front Nutr. 2023 Dec 7;10:1275291. doi: 10.3389/fnut.2023.1275291 (PMC10734639; doi:10.3389/fnut.2023.1275291)
Supplement: Supplementary file 4 [file Data_Sheet_1.docx]

**Supplementary Table 1** Global trends for age-standardized rates (per 100,000 population) of ID among children aged 0 to 14 years from 1990 to 2019

| ASPR |  |  | Age-standardized DALY rate |  |  |
| --- | --- | --- | --- | --- | --- |
| Sex | Year | APC%_95%CI | Sex | Year | APC%_95%CI |
| Both | 1990-1994 | -0.10 (-0.14 to -0.07) | Both | 1990-1994 | -0.18 (-0.25 to -0.12) |
| Both | 1994-2001 | 0.02 (0.01 to 0.04) | Both | 1994-2001 | 0.01 (-0.02 to 0.05) |
| Both | 2001-2006 | -0.10 (-0.13 to -0.07) | Both | 2001-2010 | -0.27 (-0.3 to -0.25) |
| Both | 2006-2009 | -0.28  (-0.38 to -0.17) | Both | 2010-2014 | -0.10 (-0.2 to 0.01) |
| Both | 2009-2015 | -0.05 (-0.07 to -0.02) | Both | 2014-2017 | -0.51 (-0.72 to -0.3) |
| Both | 2015-2019 | -0.52 (-0.56 to -0.48) | Both | 2017-2019 | -1.13 (-1.34 to -0.93) |
| Girl | 1990-1995 | -0.16 (-0.2 to -0.12) | Girl | 1990-1994 | -0.33 (-0.39 to -0.26) |
| Girl | 1995-2006 | -0.05 (-0.06 to -0.04) | Girl | 1994-2001 | -0.13 (-0.17 to -0.09) |
| Girl | 2006-2009 | -0.24 (-0.38 to -0.1) | Girl | 2001-2009 | -0.24 (-0.26 to -0.21) |
| Girl | 2009-2015 | -0.04 (-0.08 to -0.01) | Girl | 2009-2014 | -0.07 (-0.13 to 0) |
| Girl | 2015-2019 | -0.57 (-0.62 to -0.52) | Girl | 2014-2017 | -0.48 (-0.69 to -0.26) |
| Boy | 1990-1994 | -0.01 (-0.04 to 0.01) | Girl | 2017-2019 | -1.22 (-1.44 to -1.01) |
| Boy | 1994-2001 | 0.10 (0.09 to 0.12) | Boy | 1990-1994 | -0.03 (-0.1 to 0.04) |
| Boy | 2001-2006 | -0.14 (-0.17 to -0.12) | Boy | 1994-2001 | 0.16 (0.12 to 0.19) |
| Boy | 2006-2009 | -0.31 (-0.39 to -0.24) | Boy | 2001-2010 | -0.32 (-0.34 to -0.29) |
| Boy | 2009-2015 | -0.05 (-0.07 to -0.04) | Boy | 2010-2013 | -0.15 (-0.37 to 0.07) |
| Boy | 2015-2019 | -0.47 (-0.5 to -0.45) | Boy | 2013-2016 | -0.34 (-0.56 to -0.11) |
|  |  |  | Boy | 2016-2019 | -0.93 (-1.04 to -0.82) |

**Supplementary Table 2** Global trends for age-standardized rates (per 100,000 population) of ID among children younger than 5 years old from 1990 to 2019

| ASPR |  |  | Age-standardized DALY rate | |  |
| --- | --- | --- | --- | --- | --- |
| Sex | Year | APC%_95%CI | Sex | Year | APC%_95%CI |
| Both | 1990-1992 | -0.04  (-0.32 to 0.25) | Both | 1990-1992 | -0.16  (-0.49 to 0.17) |
| Both | 1992-1997 | 0.18  (0.11 to 0.26) | Both | 1992-1997 | 0.14  (0.04 to 0.25) |
| Both | 1997-2001 | -0.11 (-0.23 to 0.00) | Both | 1997-2001 | -0.28  (-0.45 to -0.12) |
| Both | 2001-2006 | -0.48  (-0.55 to -0.4) | Both | 2001-2010 | -0.94  (-0.98 to -0.91) |
| Both | 2006-2009 | -0.76 (-1.00 to -0.52) | Both | 2010-2015 | -0.63  (-0.74 to -0.53) |
| Both | 2009-2019 | -0.4  (-0.42 to -0.38) | Both | 2015-2019 | -0.81  (-0.92 to -0.71) |
| Girl | 1990-1992 | -0.09  (-0.34 to 0.16) | Girl | 1990-1992 | -0.32  (-0.67 to 0.03) |
| Girl | 1992-1996 | 0.19  (0.08 to 0.3) | Girl | 1992-1996 | 0.06  (-0.12 to 0.23) |
| Girl | 1996-2001 | -0.11  (-0.17 to -0.05) | Girl | 1996-2001 | -0.37  (-0.48 to -0.26) |
| Girl | 2001-2006 | -0.46  (-0.52 to -0.41) | Girl | 2001-2010 | -1  (-1.04 to -0.96) |
| Girl | 2006-2009 | -0.83 (-1.00 to -0.65) | Girl | 2010-2016 | -0.64  (-0.72 to -0.56) |
| Girl | 2009-2019 | -0.46  (-0.47 to -0.44) | Girl | 2016-2019 | -0.87  (-1.04 to -0.69) |
| Boy | 1990-1992 | -0.02  (-0.36 to 0.31) | Boy | 1990-1992 | -0.06  (-0.37 to 0.26) |
| Boy | 1992-1997 | 0.22  (0.13 to 0.31) | Boy | 1992-1997 | 0.28  (0.18 to 0.38) |
| Boy | 1997-2001 | -0.09  (-0.22 to 0.05) | Boy | 1997-2001 | -0.15  (-0.31 to 0) |
| Boy | 2001-2006 | -0.49  (-0.58 to -0.41) | Boy | 2001-2010 | -0.89  (-0.93 to -0.86) |
| Boy | 2006-2009 | -0.70 (-1.00 to -0.40) | Boy | 2010-2015 | -0.64  (-0.74 to -0.54) |
| Boy | 2009-2019 | -0.36  (-0.38 to -0.33) | Boy | 2015-2019 | -0.82  (-0.92 to -0.72) |

**Supplementary Table 3** Global trends for age-standardized rates (per 100,000 population) of ID among children aged 5-9 years from 1990 to 2019

| ASPR |  |  | Age-standardized DALY rate |  |  |
| --- | --- | --- | --- | --- | --- |
| Sex | Year | APC%_95%CI | Sex | Year | APC%_95%CI |
| Both | 1990-1996 | -0.32  (-0.36 to -0.28) | Both | 1990-1996 | -0.34  (-0.41 to -0.27) |
| Both | 1996-2001 | 0.60 (0.53 to 0.66) | Both | 1996-2001 | 0.75  (0.62 to 0.89) |
| Both | 2001-2005 | 0.20 (0.10 to 0.30) | Both | 2001-2016 | 0.03  (0.02 to 0.05) |
| Both | 2005-2009 | -0.08  (-0.19 to 0.02) | Both | 2016-2019 | -1.16  (-1.36 to -0.95) |
| Both | 2009-2015 | 0.15 (0.10 to 0.20) | Girl | 1990-1996 | -0.49  (-0.52 to -0.46) |
| Both | 2015-2019 | -0.62  (-0.69 to -0.54) | Girl | 1996-2001 | 0.64 (0.57 to 0.70) |
| Girl | 1990-1996 | -0.43  (-0.48 to -0.38) | Girl | 2001-2010 | 0.00 (-0.02 to 0.02) |
| Girl | 1996-2002 | 0.48  (0.42 to 0.54) | Girl | 2010-2014 | 0.31  (0.2 to 0.41) |
| Girl | 2002-2010 | 0.00 (-0.03 to 0.03) | Girl | 2014-2017 | -0.43  (-0.64 to -0.23) |
| Girl | 2010-2015 | 0.20 (0.11 to 0.28) | Girl | 2017-2019 | -1.40 (-1.6 to -1.2) |
| Girl | 2015-2019 | -0.70 (-0.79 to -0.62) | Boy | 1990-1996 | -0.19  (-0.24 to -0.13) |
| Boy | 1990-1996 | -0.20 (-0.24 to -0.16) | Boy | 1996-2001 | 0.85  (0.75 to 0.95) |
| Boy | 1996-2001 | 0.68  (0.62 to 0.74) | Boy | 2001-2005 | 0.12 (-0.05 to 0.28) |
| Boy | 2001-2005 | 0.25  (0.15 to 0.35) | Boy | 2005-2016 | -0.03  (-0.05 to 0) |
| Boy | 2005-2010 | -0.06  (-0.12 to 0.01) | Boy | 2016-2019 | -1.07  (-1.23 to -0.91) |
| Boy | 2010-2015 | 0.15  (0.08 to 0.21) |  |  |  |
| Boy | 2015-2019 | -0.55  (-0.62 to -0.48) |  |  |  |

**Supplementary Table 4** Global trends for age-standardized rates (per 100,000 population) of ID among children aged 10-14 years from 1990 to 2019

| ASPR |  |  | Age-standardized DALY rate |  |  |
| --- | --- | --- | --- | --- | --- |
| Sex | Year | APC%_95%CI | Sex | Year | APC%_95%CI |
| Both | 1990-1995 | -0.10 (-0.15 to -0.05) | Both | 1990-1995 | -0.13  (-0.18 to -0.07) |
| Both | 1995-2000 | -0.69  (-0.75 to -0.63) | Both | 1995-2000 | -0.66  (-0.74 to -0.59) |
| Both | 2000-2003 | -0.13  (-0.33 to 0.07) | Both | 2000-2003 | -0.31  (-0.55 to -0.06) |
| Both | 2003-2010 | 0.69  (0.65 to 0.72) | Both | 2003-2010 | 0.75  (0.71 to 0.79) |
| Both | 2010-2016 | 0.30 (0.25 to 0.35) | Both | 2010-2016 | 0.18  (0.13 to 0.24) |
| Both | 2016-2019 | -0.71  (-0.84 to -0.58) | Both | 2016-2019 | -1.09  (-1.21 to -0.96) |
| Girl | 1990-1995 | -0.22  (-0.29 to -0.16) | Girl | 1990-1995 | -0.26  (-0.32 to -0.2) |
| Girl | 1995-2000 | -0.76  (-0.84 to -0.68) | Girl | 1995-2000 | -0.73  (-0.81 to -0.64) |
| Girl | 2000-2003 | 0.04 (-0.20 to 0.28) | Girl | 2000-2003 | -0.13  (-0.4 to 0.14) |
| Girl | 2003-2010 | 0.83  (0.79 to 0.87) | Girl | 2003-2010 | 0.9  (0.86 to 0.95) |
| Girl | 2010-2016 | 0.3  (0.24 to 0.35) | Girl | 2010-2016 | 0.16  (0.1 to 0.22) |
| Girl | 2016-2019 | -0.73  (-0.87 to -0.58) | Girl | 2016-2019 | -1.13  (-1.27 to -0.99) |
| Boy | 1990-1995 | 0.1  (0.06 to 0.15) | Boy | 1990-1995 | 0.09  (0.03 to 0.14) |
| Boy | 1995-2000 | -0.57  (-0.63 to -0.51) | Boy | 1995-2003 | -0.53  (-0.56 to -0.49) |
| Boy | 2000-2003 | -0.31 (-0.50 to -0.11) | Boy | 2003-2011 | 0.53  (0.5 to 0.57) |
| Boy | 2003-2011 | 0.48  (0.45 to 0.51) | Boy | 2011-2016 | 0.14  (0.05 to 0.22) |
| Boy | 2011-2016 | 0.26  (0.19 to 0.34) | Boy | 2016-2019 | -1  (-1.13 to -0.88) |
| Boy | 2016-2019 | -0.67  (-0.8 to -0.55) |  |  |  |

**Supplementary Table** 5 Prevalence and DALY of global children with ID in different age groups in 1990 and 2019 and the AAPC

|  | 1990 |  | 2019 |  | 1990-2019 |
| --- | --- | --- | --- | --- | --- |
|  | Cases NO.(95%UI) | ASR/100,000 (95% CI) | Cases NO.(95%UI) | ASR/100,000 (95% CI) | AAPC% (95%CI) |
| Prevalence | |  |  |  |  |
| Both |  |  |  |  |  |
| <5 years | 184219912.17 (179938814.63 to 188529508.34) | 29143.37 (28466.1 to 29825.14) | 177639145.19 (173277823.08 to 181900503.82) | 26799.59 (26141.62 to 27442.49) | -0.29  (-0.32 to -0.25) |
| 5–9 years | 122191657.5 (117171397.5 to 127286674.21) | 20881.67 (20023.74 to 21752.37) | 136523297.68 (130935947.62 to 141959361.4) | 20852.68 (19999.27 to 21682.99) | 0 (-0.03 to 0.03) |
| 10–14 years | 64610047.72 (61167740.19 to 68002226.98) | 12038.63 (11397.23 to 12670.68) | 77329256.36 (72800653.25 to 82198083.91) | 12041.55 (11336.37 to 12799.72) | 0 (-0.03 to 0.03) |
| Boy |  |  |  |  |  |
| <5 years | 96471068.97 (93430282.72 to 99837435.24) | 29647.78 (28713.27 to 30682.34) | 94190082.13 (90787688.12 to 97497396.49) | 27508.89 (26515.19 to 28474.81) | -0.26  (-0.3 to -0.21) |
| 5–9 years | 62419679.29 (58687073.59 to 66421143.91) | 20769.94 (19527.93 to 22101.42) | 71123405.34 (66783897.51 to 75892445.89) | 21045.59 (19761.52 to 22456.76) | 0.05 (0.03 to 0.07) |
| 10–14 years | 28079876.62 (25696583.18 to 30512247) | 10219.38 (9352.01 to 11104.62) | 33783801.75 (30361960.98 to 37428493.45) | 10196.29 (9163.55 to 11296.3) | 0 (-0.03 to 0.02) |
| Girl |  |  |  |  |  |
| <5 years | 87748843.19 (85079845.89 to 90340370.14) | 28608.26 (27738.1 to 29453.16) | 83449063.06 (81263123.82 to 85730733.5) | 26041.7 (25359.55 to 26753.74) | -0.32  (-0.35 to -0.29) |
| 5–9 years | 59771978.21 (56862454.59 to 62780270.57) | 20999.63 (19977.43 to 22056.53) | 65399892.34 (62149665.35 to 68546647.55) | 20646.87 (19620.77 to 21640.31) | -0.05 (-0.08 to -0.03) |
| 10–14 years | 36530171.1 (34160104.84 to 38889718.82) | 13947.14 (13042.25 to 14848.01) | 43545454.61 (40611694.21 to 46491839.92) | 14008.4 (13064.62 to 14956.24) | 0.02 (-0.02 to 0.05) |
| DALY |  |  |  |  |  |
| Both |  |  |  |  |  |
| <5 years | 5781575.45 (3849030.82 to 8434650.87) | 914.64 (608.91 to 1334.35) | 5179894.79 (3456696.26 to 7543559.19) | 781.47 (521.5 to 1138.06) | -0.54  (-0.58 to -0.5) |
| 5–9 years | 4918979.23 (3268494.37 to 7195100.06) | 840.62 (558.56 to 1229.59) | 5432574.74 (3643632.62 to 7876613.37) | 829.78 (556.53 to 1203.08) | -0.04 (-0.08 to -0.01) |
| 10–14 years | 2564780.13 (1689352.76 to 3678269.9) | 477.89 (314.77 to 685.36) | 3007761.11 (1990937.62 to 4365502.59) | 468.36 (310.02 to 679.79) | -0.06 (-0.1 to -0.03) |
| Boy |  |  |  |  |  |
| <5 years | 2980367.77 (1989180.54 to 4347846.64) | 915.94 (611.32 to 1336.19) | 2730450.68 (1813138.62 to 4042290) | 797.45 (529.54 to 1180.58) | -0.48  (-0.52 to -0.44) |
| 5–9 years | 2506302.72 (1665636.07 to 3655393.79) | 833.96 (554.23 to 1216.32) | 2818038.84 (1848692.67 to 4151601.97) | 833.86 (547.03 to 1228.47) | 0 (-0.03 to 0.03) |
| 10–14 years | 1100006.63 (710166.92 to 1584407.96) | 400.34 (258.46 to 576.63) | 1298958.15 (840477.57 to 1870878.89) | 392.04 (253.66 to 564.65) | -0.07 (-0.09 to -0.04) |
| Girl |  |  |  |  |  |
| <5 years | 2801207.68 (1858755.18 to 4066532.65) | 913.26 (606 to 1325.79) | 2449444.1 (1622756.12 to 3574801.95) | 764.39 (506.41 to 1115.58) | -0.61 (-0.65 to -0.57) |
| 5–9 years | 2412676.52 (1608779.84 to 3480904.69) | 847.64 (565.21 to 1222.94) | 2614535.9 (1744186.84 to 3774506.81) | 825.41 (550.64 to 1191.62) | -0.09 (-0.12 to -0.06) |
| 10–14 years | 1464773.5 (972801.46 to 2093694.79) | 559.25 (371.41 to 799.37) | 1708802.97 (1133791.49 to 2496069.82) | 549.72 (364.74 to 802.98) | -0.05 (-0.09 to -0.02) |

**Supplementary Table 6** Prevalence of ID among children in 1990 and 2019 and the EAPC from 1990 to 2019 in 204 countries and territories

| Location | 1990 |  | 2019 |  | 1990-2019 |
| --- | --- | --- | --- | --- | --- |
|  | Prevalence cases NO.(95%UI) | ASR/100,000 (95% CI) | Prevalence cases NO.(95%UI) | ASR/100,000 (95% CI) | EAPC (95%CI) |
| Bhutan | 121070.95 (81876.5 to 140871.26) | 47546.52 (31029.32 to 57420.59) | 104878.29 (95773.05 to 114486.75) | 54729.81 (46877.72 to 62083.13) | 0.33 (0.21 to 0.44) |
| Mali | 1591654.15 (1453872.49 to 1727696.42) | 37609.63 (31441.31 to 43240.95) | 4973383.24 (4438105.12 to 5427161.4) | 47477.39 (40291 to 54123.33) | 0.85 (0.71 to 0.99) |
| Gambia | 215121.47 (198484.82 to 230730.32) | 44439.39 (38485.44 to 50291.08) | 421389.32 (380958.42 to 459974.7) | 45473.78 (38237.83 to 52214.25) | -0.14 (-0.22 to -0.05) |
| Burkina Faso | 1459119.55 (1269455.36 to 1648421.71) | 29783.32 (23608.87 to 36051.48) | 4515769.31 (3850910.14 to 5230677.55) | 42571.67 (34401.56 to 51755.86) | 1.43 (1.31 to 1.55) |
| Senegal | 1581340.92 (1438337.61 to 1721043.27) | 42418.65 (35297.66 to 48982.29) | 2525796.9 (2263915.81 to 2763122.22) | 41326.88 (34771.48 to 47745.3) | -0.13 (-0.22 to -0.03) |
| Zambia | 1424571.72 (1284181.3 to 1564117.39) | 37466.04 (31152.04 to 43295.87) | 3092519.7 (2804897.03 to 3403986.32) | 39915.22 (33079.97 to 46753.43) | 0.3 (0.24 to 0.35) |
| Nigeria | 15211567.22 (14129347.18 to 16423196.6) | 37712.52 (33307.54 to 42161.95) | 36200361.89 (32278522.76 to 40187726.47) | 38450.1 (31859.96 to 44894.8) | 0.07 (0 to 0.14) |
| Sierra Leone | 519809.15 (434910.38 to 602233.81) | 31595.32 (23708.5 to 39045.75) | 1243960.31 (1117960.36 to 1365777.37) | 36908.95 (31064.82 to 42493.48) | 0.59 (0.56 to 0.63) |
| Guinea-Bissau | 149210.18 (125674.06 to 175565.43) | 30222.58 (22406.83 to 37750.22) | 294813.48 (255255.51 to 334694.05) | 36719.6 (28244.77 to 45197.52) | 0.89 (0.76 to 1.02) |
| Togo | 592021.5 (513539.03 to 672727.83) | 32255.26 (26073.06 to 38845.23) | 1157904.52 (1034327.25 to 1281944.79) | 36565.83 (30095.85 to 42989.7) | 0.4 (0.22 to 0.57) |
| Mauritania | 378773.01 (337235.3 to 420372.02) | 38974.15 (31249.57 to 46701.01) | 581363.06 (411149.54 to 678375.69) | 36475.63 (24297.75 to 45714.11) | -0.05 (-0.1 to 0) |
| Chad | 1054619.1 (928701.46 to 1189130.13) | 34371.86 (27081.01 to 41664.14) | 3107428.45 (2702901.42 to 3514501.33) | 36197.67 (28214.01 to 44202.28) | 0.15 (0.12 to 0.18) |
| Niger | 1502030.4 (1284725.86 to 1682979.76) | 35823.75 (27943.31 to 42848.83) | 4458157.18 (3851685.32 to 5031147.95) | 36147.24 (28143.3 to 43318.04) | -0.06 (-0.17 to 0.06) |
| Haiti | 766931.41 (655769.38 to 871813.5) | 28034.39 (21374.07 to 34991.72) | 1473440.47 (1287766.01 to 1684692.34) | 34337.94 (26694.74 to 42298.22) | 0.91 (0.85 to 0.96) |
| Yemen | 1943068.09 (1724507.5 to 2143707.44) | 24791.91 (20746.81 to 29176.45) | 4327698.39 (3883638.21 to 4755819.08) | 33594.07 (28665.26 to 38997.44) | 1.36 (1.22 to 1.5) |
| Malawi | 1548242.41 (1359556.72 to 1734994.69) | 34086.38 (27170.27 to 40996.37) | 2612734.1 (2292609.15 to 2959587.26) | 33532.49 (26616.85 to 40711.66) | -0.2 (-0.32 to -0.08) |
| United Republic of Tanzania | 4696793.67 (4278648.47 to 5096468.43) | 37841.17 (31886.75 to 43388.05) | 8342556.23 (7266336.36 to 9490628.27) | 33532.13 (25793.05 to 41444.52) | -0.6 (-0.71 to -0.49) |
| India | 119863461.78 (116005111.5 to 123434804.8) | 35849.6 (34128.96 to 37522.18) | 121283812.6 (117269465.97 to 125197595.32) | 32886.47 (31275.54 to 34514.7) | -0.3 (-0.36 to -0.24) |
| Cabo Verde | 53696.72 (46394.99 to 61935.34) | 33766.98 (25369.75 to 42346.66) | 49939.17 (41059.79 to 58684.91) | 31816.97 (22598.77 to 42289.94) | -0.25 (-0.3 to -0.19) |
| Gabon | 136706.12 (114630.85 to 158854.21) | 32068.06 (23297.7 to 40703.28) | 184834.62 (145553.71 to 220872.71) | 31809.81 (23060.68 to 40897.81) | 0.08 (-0.1 to 0.26) |
| Central African Republic | 364826.95 (282406.94 to 437295.56) | 28290.19 (19418.44 to 36446.58) | 715171.11 (558099.76 to 858512.75) | 31378.09 (21874.91 to 40556.76) | 0.3 (0.22 to 0.39) |
| Pakistan | 16556704.96 (14423500.53 to 18613955.09) | 32089.95 (24519.63 to 39754.36) | 27331933.21 (23207094.38 to 31346254.21) | 31365.16 (23774 to 39462.62) | -0.2 (-0.33 to -0.08) |
| Guinea | 787233.72 (697876.94 to 883306.86) | 25558.41 (20231.41 to 31206.87) | 1817457.17 (1590099.09 to 2041415.41) | 30985.95 (24496.32 to 37209.25) | 0.76 (0.69 to 0.82) |
| Mozambique | 1915353.76 (1671127.85 to 2150062.7) | 30689.49 (24265.55 to 36877.77) | 4347329.19 (3813104.87 to 4947664.2) | 30886.64 (24672.69 to 37352.42) | 0.09 (0.05 to 0.14) |
| Myanmar | 4866077.63 (4115711.62 to 5613300.02) | 30535.72 (22581.16 to 38994.79) | 4531392.75 (3887993.61 to 5219367.5) | 30793.06 (23590.07 to 38854.46) | 0.05 (0.03 to 0.08) |
| Eritrea | 494830.03 (432729.72 to 554765.53) | 34504.52 (26963.92 to 42153.72) | 803105.85 (685260.26 to 920700.87) | 30356.11 (22151.2 to 38781.59) | -0.54 (-0.58 to -0.5) |
| South Sudan | 816750.46 (693290.34 to 937730.72) | 30820.6 (23334.34 to 38475.42) | 1247821 (1018700.71 to 1479908.13) | 29777.39 (21270.34 to 38403.1) | -0.19 (-0.24 to -0.15) |
| Ghana | 1781552.13 (1525052.63 to 2031001.46) | 24761.98 (19530.36 to 30421.83) | 3367207.36 (2867016.14 to 3905879.17) | 29757.2 (22720.15 to 37408.22) | 0.71 (0.6 to 0.82) |
| Sao Tome and Principe | 16419.97 (9483.48 to 20300.88) | 28404.68 (15254.06 to 38952.22) | 21328.33 (16773.56 to 25972.26) | 29619.59 (19671.4 to 39321.92) | 0.65 (-0.01 to 1.31) |
| Comoros | 71295.32 (45250.14 to 82965.61) | 33128.41 (20175 to 42022.23) | 68145.78 (52578.57 to 81284.63) | 29535 (20133.28 to 38796.72) | -0.58 (-0.69 to -0.47) |
| Somalia | 1117213.49 (978782.51 to 1269657.43) | 31929.01 (25060.93 to 39003.46) | 2865169.08 (2496866.89 to 3243283.15) | 29526.99 (22727.4 to 36842.17) | -0.32 (-0.34 to -0.29) |
| Djibouti | 73800.89 (63185.11 to 84368.66) | 32781.47 (24958.08 to 41394.98) | 121254.07 (98431.12 to 144386.77) | 28283.06 (19718.1 to 37333.65) | -0.63 (-0.71 to -0.55) |
| Congo | 291523.23 (241543.27 to 347297.66) | 26156.78 (18843.47 to 33991.21) | 558528.56 (435946.25 to 673420.88) | 27987.6 (19362.62 to 36567.7) | 0.32 (0.25 to 0.38) |
| Cambodia | 1483235.84 (1273640.88 to 1693612.96) | 29899.51 (22538.81 to 37632.29) | 1403803.99 (1159575.81 to 1638148.89) | 27851.39 (20342.03 to 36026.13) | -0.21 (-0.28 to -0.14) |
| Benin | 650229.08 (554027.08 to 747860.39) | 25221.61 (19462.54 to 31096.28) | 1618634.6 (1384199.75 to 1876148.36) | 27457.27 (20377.15 to 34782.75) | 0.16 (-0.02 to 0.33) |
| Democratic Republic of the Congo | 5339032.63 (4607958.76 to 6060582) | 28329.88 (21827.14 to 34916) | 10426201.64 (8856673.37 to 12024187.12) | 27423.8 (21045.36 to 34123.07) | -0.16 (-0.26 to -0.05) |
| Nepal | 2334658.81 (1969705.49 to 2690287.63) | 26121.68 (19529.62 to 33121.55) | 2446948.78 (2057563.44 to 2867376.05) | 27268.15 (20228.69 to 35477.9) | -0.09 (-0.19 to 0.01) |
| Ethiopia | 7164027.28 (6696264.2 to 7668005.22) | 27798.63 (24590.12 to 31042.94) | 12372264.61 (11502857.93 to 13312047.33) | 26549.87 (23490.64 to 29826.64) | -0.21 (-0.25 to -0.17) |
| Uzbekistan | 2657638.78 (2295756.97 to 3042200.72) | 30012.62 (23076.42 to 37768.33) | 2700612.72 (2205816.85 to 3204049.33) | 26080.17 (18589.77 to 34837.01) | -0.52 (-0.59 to -0.45) |
| Burundi | 704675.41 (571525.74 to 833997.82) | 25363 (18071.67 to 33216.07) | 1412200.17 (1196279.71 to 1655898.7) | 25408.18 (19193.44 to 32223.04) | 0.44 (0.3 to 0.59) |
| Cameroon | 1111356.42 (939593.72 to 1282563.44) | 21938.98 (16528.59 to 27654.44) | 3009923.95 (2488477.71 to 3573275.37) | 24950.09 (18426.04 to 32141.57) | 0.51 (0.38 to 0.64) |
| Madagascar | 1754016.36 (1518531.77 to 1989898.21) | 30673.81 (23807.64 to 38193.51) | 2722797.28 (2292544.63 to 3141426.11) | 24748.19 (18420.5 to 31618) | -0.84 (-0.99 to -0.69) |
| Lesotho | 166611.55 (140952.08 to 194759.15) | 21827.43 (15937.73 to 28264.11) | 158061.2 (136118.5 to 182815.84) | 24366.55 (18642.56 to 30483.04) | 0.52 (0.43 to 0.61) |
| Timor-Leste | 83153.23 (40591.52 to 107480.8) | 23127.69 (9958.5 to 33211.21) | 120343.88 (98960.24 to 141269.5) | 23971 (17442.2 to 31616) | -0.16 (-0.29 to -0.03) |
| Equatorial Guinea | 59313.61 (49229.51 to 69136.81) | 27774.21 (19947.38 to 35752.61) | 132217.46 (99265.19 to 170134.85) | 23870.1 (15257.32 to 33378.77) | -0.66 (-0.76 to -0.56) |
| Namibia | 167647.37 (119141.52 to 198458.56) | 27756.53 (18650.81 to 35612.01) | 198093.74 (155120.07 to 230598.92) | 23533.21 (16499.25 to 30407.9) | -0.6 (-0.72 to -0.48) |
| Liberia | 256409.59 (206190.24 to 304626.94) | 27604.76 (20249.62 to 34793) | 432371.34 (340131.68 to 530110.66) | 23400.87 (16179.96 to 30949.96) | -0.43 (-0.51 to -0.35) |
| Papua New Guinea | 405741.63 (345268.15 to 468532.73) | 23617.89 (17657.01 to 29825.01) | 878407.54 (738895.53 to 1020470.52) | 23033.79 (16955.27 to 29681.01) | 0.03 (-0.01 to 0.07) |
| Guyana | 77432.48 (64565.65 to 91523.94) | 27187.35 (19664.58 to 35444.63) | 47611.84 (37866.2 to 57795.68) | 22982.23 (15561.88 to 31264.76) | -0.37 (-0.43 to -0.31) |
| Botswana | 154439.76 (132857.04 to 177374.92) | 27034.52 (21065.44 to 34086.17) | 160384.41 (126345.1 to 195069.73) | 22892.28 (15188.91 to 31801.73) | -0.56 (-0.64 to -0.48) |
| Zimbabwe | 927011.98 (749634.14 to 1107247.02) | 18876.29 (12840.85 to 26073) | 1357990.6 (1135212.32 to 1590307.76) | 22657.24 (16465.83 to 29542.04) | 0.84 (0.63 to 1.05) |
| Marshall Islands | 5806.43 (5242.75 to 6393.75) | 25870.73 (21751 to 30656.85) | 4057.41 (3336.5 to 4835.4) | 22545.8 (16552.07 to 29682.36) | -0.51 (-0.57 to -0.45) |
| Fiji | 59386 (50761.08 to 68633.05) | 21201.73 (15914.22 to 26852.17) | 58354.41 (47437.9 to 69833.18) | 22147.66 (15163.4 to 29554.05) | 0.31 (0.22 to 0.4) |
| Angola | 791117.03 (649485.61 to 947638.9) | 15982.47 (11121.66 to 21501.39) | 3163127.3 (2627792.51 to 3787633.4) | 22108.1 (15928.42 to 29531.69) | 1.55 (1.4 to 1.69) |
| Kiribati | 7260.03 (6172.93 to 8440.66) | 23786.26 (17794.67 to 30328.45) | 9147.23 (7535.42 to 10981.19) | 21769.3 (15467.57 to 29009.13) | -0.31 (-0.34 to -0.27) |
| Uganda | 2380018.13 (2081121.31 to 2695221.66) | 26860.87 (20771.8 to 33180.47) | 4193953.72 (3554629.08 to 4914136.19) | 21655.41 (15837.91 to 27796.76) | -0.68 (-0.85 to -0.51) |
| Sudan | 2334134.93 (1969999.24 to 2704850.78) | 24627.97 (17924.55 to 31406.08) | 3397121.19 (2853252.77 to 4001881.83) | 21524.17 (15893.77 to 27820.5) | -0.34 (-0.38 to -0.3) |
| Solomon Islands | 36443.18 (29731.82 to 42867.71) | 22473.89 (16094.52 to 29264.41) | 56717.55 (45777.01 to 68034.41) | 21522.96 (14778.62 to 29009.77) | -0.12 (-0.16 to -0.09) |
| Bolivia (Plurinational State of) | 564609.45 (476405.84 to 652009.62) | 20489.29 (15269.3 to 26088.57) | 867689.82 (721529.54 to 1011724.8) | 21236.83 (15603.26 to 27883.6) | 0.51 (0.37 to 0.65) |
| Bangladesh | 15629964.48 (13552924.44 to 17786436.92) | 31116.49 (24072.7 to 38591.56) | 8938247.76 (7182202.85 to 10870321.49) | 21223.25 (14838.87 to 29076.25) | -1.51 (-1.6 to -1.43) |
| Grenada | 7949.47 (7001.59 to 8904.75) | 24990.28 (20099.88 to 30045.5) | 4455.38 (3396.44 to 5594.79) | 20898.98 (13693.8 to 29604.31) | -0.53 (-0.59 to -0.46) |
| Belize | 18293.93 (14449.49 to 22506.09) | 22492.79 (15047.49 to 31197.05) | 24439.75 (18821.09 to 30725.87) | 20761.24 (12935.06 to 29974.21) | -0.26  (-0.32 to -0.2) |
| Suriname | 29441.89 (23132.92 to 36253.41) | 22609.68 (14776.86 to 31395.08) | 29340.1 (22551.9 to 36510.86) | 20585.33 (12957.53 to 29809.57) | -0.27  (-0.29 to -0.25) |
| Saint Vincent and the Grenadines | 8616.95 (6736.12 to 10834.92) | 21135.17 (13153.18 to 30180.53) | 4810.27 (3663.88 to 6111.86) | 19953.66 (12117.05 to 28862.04) | -0.2  (-0.27 to -0.13) |
| Eswatini | 78316.53 (63145.76 to 93575.05) | 19817.14 (13413.27 to 26657.56) | 78530.25 (63174.57 to 94693.62) | 19294.94 (13120.7 to 26673.65) | 0.01  (-0.11 to 0.13) |
| Micronesia (Federated States of) | 11347.21 (9950.23 to 12718.91) | 23875.77 (19011.96 to 28916.79) | 5615.95 (4488.29 to 6797.74) | 18982.11 (12852.43 to 25939.89) | -0.79  (-0.86 to -0.71) |
| Tuvalu | 786.89 (651.79 to 935.34) | 22542.09 (15790.35 to 30126.3) | 619.79 (481.86 to 761.7) | 18933.23 (12539.46 to 26187.18) | -0.53  (-0.58 to -0.48) |
| Kyrgyzstan | 369699.59 (304725.84 to 434855.34) | 21648.73 (15589.07 to 28738.34) | 398550.65 (321608.63 to 482833.53) | 18724.62 (12929.1 to 25740.34) | -0.52  (-0.61 to -0.43) |
| Oman | 283547.96 (255335.04 to 312050.08) | 32657.33 (27009.98 to 38222.63) | 202715.29 (149005.53 to 263791.06) | 18686.62 (10940.93 to 28236.55) | -1.95  (-2.14 to -1.75) |
| Mauritius | 79524.01 (66936.83 to 93672.03) | 25304.41 (19055.28 to 32330.84) | 36488.01 (27950.69 to 45463.43) | 18623.74 (12299.02 to 26305.97) | -1  (-1.05 to -0.95) |
| Saint Lucia | 11644.51 (9127.61 to 14496.96) | 22207.6 (14582.6 to 31168.78) | 5556.94 (4152.43 to 7137.27) | 18522.53 (10976.39 to 27709.56) | -0.6  (-0.69 to -0.52) |
| Rwanda | 730541.62 (605898.16 to 851092.22) | 20784.92 (15400.78 to 26730.5) | 886900.62 (702151.64 to 1069651.43) | 18450.26 (12773.01 to 24807.64) | -0.29  (-0.45 to -0.12) |
| Trinidad and Tobago | 86680.6 (67879.97 to 107833.61) | 21629.81 (13890.69 to 30673.11) | 48185.77 (35778.16 to 62441.9) | 18399.2 (10745.36 to 27509.26) | -0.64  (-0.71 to -0.57) |
| Antigua and Barbuda | 4279.18 (3357.64 to 5169.41) | 22906.92 (15195.1 to 31486.18) | 2916.69 (2190.22 to 3698.67) | 18343.66 (10737.71 to 27087.19) | -0.87  (-0.93 to -0.8) |
| Jamaica | 167695.81 (136955.8 to 201814.32) | 20525.29 (14527.53 to 27899.44) | 103347.9 (76622.65 to 131808.9) | 17983.26 (11530.63 to 26332.89) | -0.5  (-0.6 to -0.39) |
| Maldives | 36626.98 (32167.29 to 41175.66) | 33267.44 (26594.78 to 40691.2) | 20253 (15168.73 to 25504.98) | 17719.64 (10950.39 to 26020.93) | -2.67  (-2.86 to -2.49) |
| Lao People's Democratic Republic | 436442.47 (364963.14 to 510141.62) | 23331.06 (16476.16 to 31026.86) | 398354.49 (311406.42 to 491198.72) | 17639.16 (11373.55 to 25014.93) | -1.09  (-1.16 to -1.02) |
| Nauru | 982.83 (794.85 to 1179.71) | 20579.31 (14208.46 to 28157.88) | 697.38 (550.41 to 880.86) | 17622.25 (11420.57 to 25198.77) | -0.43  (-0.52 to -0.33) |
| Bahamas | 15698.9 (12050.23 to 19807.67) | 20021.47 (12710.86 to 28876.52) | 12974.8 (9563.12 to 16718.06) | 17568.88 (10289.56 to 26501.73) | -0.41  (-0.52 to -0.31) |
| Guatemala | 664945.66 (552883.58 to 779903.47) | 17594.75 (12536.86 to 23191.19) | 1010352.23 (826319.53 to 1221177.61) | 17511.19 (12063.57 to 23570.99) | 0.22  (0.03 to 0.42) |
| Tokelau | 165.22 (137.8 to 197.94) | 21944.7 (15063.7 to 29657.71) | 83.85 (65.06 to 102.88) | 17282.19 (11167.15 to 24643.93) | -0.82  (-0.84 to -0.79) |
| Samoa | 12082.62 (9704 to 14759.93) | 19029.94 (13289.98 to 25817.75) | 11739.35 (9111.29 to 14875.15) | 17072.77 (11096.1 to 24143.82) | -0.28  (-0.38 to -0.19) |
| Peru | 1992640.63 (1679837.36 to 2363368.35) | 23595.23 (17152.69 to 30703.78) | 1536314.51 (1198598.58 to 1890078.19) | 16976.38 (11284.69 to 23965.17) | -1.29  (-1.43 to -1.16) |
| Vanuatu | 9380.19 (7253.76 to 11664.88) | 13316.47 (8678 to 18824.4) | 17768.52 (13931.95 to 21638.38) | 16154.61 (10757 to 22199.75) | 0.39  (0.26 to 0.53) |
| Barbados | 11945.06 (9707.88 to 14453.27) | 19160.78 (13205.3 to 26000.22) | 7792.67 (5806.27 to 10119.16) | 16147.58 (9603.3 to 24675.84) | -0.57  (-0.62 to -0.52) |
| Morocco | 2022000.05 (1624285.82 to 2448715.54) | 20436.07 (13933.23 to 28053.11) | 1468390.73 (1106619.86 to 1865773.16) | 16013.7 (9967.75 to 23553.59) | -0.75  (-0.79 to -0.72) |
| Egypt | 4923665.35 (4021563.69 to 5884182.82) | 21813.26 (15104.52 to 29294.05) | 5195499.93 (3947890.82 to 6562593.65) | 15928.19 (10105.24 to 22994.66) | -0.82  (-0.99 to -0.66) |
| Guam | 7591.61 (5870.12 to 9426.11) | 17369.14 (11292.04 to 24948.29) | 7086.97 (5401.12 to 9178.75) | 15584.5 (9692.5 to 23083.92) | -0.17  (-0.34 to 0) |
| Saint Kitts and Nevis | 2666.21 (1980.49 to 3387.66) | 19289.8 (11972.07 to 28061.09) | 1746.35 (1260.45 to 2291.15) | 15531.89 (8730.01 to 23992.48) | -0.7  (-0.8 to -0.6) |
| Honduras | 430921.81 (363824.92 to 503852.39) | 19100.64 (13858.9 to 24896.92) | 506648.99 (400977.06 to 625790.89) | 15520.99 (10612.06 to 21980.4) | -0.63  (-0.69 to -0.57) |
| United States Virgin Islands | 5814.07 (4363.41 to 7254.66) | 18482.28 (11448.42 to 26980.52) | 3002.42 (2189.08 to 3910.03) | 15468.94 (8588.69 to 24475.2) | -0.69  (-0.83 to -0.55) |
| Kazakhstan | 1084685.03 (940791.22 to 1231421.83) | 20804.45 (16226.6 to 25886.51) | 798812.95 (629776.93 to 979809.74) | 15451.48 (10051.6 to 21907.72) | -1.24  (-1.31 to -1.17) |
| Tajikistan | 457745.07 (348410.03 to 545993.06) | 18850.54 (12439.47 to 25535.87) | 497012.9 (387852.36 to 604930.22) | 14996.31 (9827.47 to 21048.36) | -0.98  (-1.05 to -0.9) |
| Palau | 859.17 (656.58 to 1051.94) | 18248.71 (12098.37 to 25816.53) | 463.17 (343.33 to 598.5) | 14966.44 (9068.24 to 22539.61) | -0.6  (-0.69 to -0.52) |
| Niue | 158.21 (124.38 to 196.76) | 19161.19 (12788 to 26675.8) | 56.69 (42.64 to 73.16) | 14881.68 (9050.34 to 22194.87) | -0.91  (-0.98 to -0.85) |
| Afghanistan | 878200.67 (717427.27 to 1046713.79) | 17040.77 (11818.13 to 23060.16) | 2628460.43 (2150936.72 to 3176582.04) | 14764.18 (10104.02 to 19907.51) | -0.51  (-0.56 to -0.46) |
| Georgia | 234991.88 (181751.23 to 291742.35) | 17331.53 (11165.26 to 24520.27) | 106319.26 (80041.18 to 134545.63) | 14667.16 (9048.85 to 21593.81) | -0.48  (-0.56 to -0.4) |
| Kenya | 1871504.61 (1761772.22 to 1978297.69) | 16015.74 (14698.99 to 17323.41) | 2727076 (2507228.62 to 2926005.11) | 14470.15 (12991.68 to 15958.16) | -0.37  (-0.45 to -0.3) |
| Turkmenistan | 289192.5 (241950.99 to 340700.37) | 18548.73 (13438.77 to 24524.03) | 222745.58 (175517.51 to 278984.33) | 14396.63 (9355.66 to 20597.54) | -0.96  (-1.03 to -0.89) |
| Tonga | 6232.75 (4859.42 to 7625.32) | 16031.57 (10709.91 to 21816.42) | 5066.57 (3870.82 to 6461.5) | 14339.02 (9105.97 to 20372.46) | -0.33  (-0.38 to -0.28) |
| Syrian Arab Republic | 1211836.86 (955909.04 to 1487510.39) | 19402.75 (12948.06 to 27212.21) | 517866.79 (368502.08 to 682705.8) | 14231.13 (8176.85 to 21562.53) | -1.14  (-1.29 to -0.99) |
| Cuba | 438863.18 (317244.34 to 562773.9) | 17524.98 (10610.06 to 26334.74) | 247934.87 (176384.6 to 330606.55) | 14200.4 (7739.25 to 22597.12) | -0.74  (-0.82 to -0.66) |
| Armenia | 160447.43 (133170.26 to 191979.99) | 15160.86 (10608.87 to 20458.86) | 85662.95 (68039.12 to 104567.58) | 14184.21 (9484.79 to 19825.25) | -0.07  (-0.16 to 0.03) |
| Seychelles | 4558.9 (3586.52 to 5620.83) | 19290.12 (12690.16 to 27292.93) | 3022.94 (2180.9 to 3991.95) | 14000.17 (8123.82 to 21780.95) | -1.15  (-1.21 to -1.1) |
| Northern Mariana Islands | 2216.79 (1707.2 to 2750.14) | 16873.72 (10827.37 to 24127.06) | 947.31 (701.32 to 1229.23) | 13911.57 (8691.78 to 20663.9) | -0.45  (-0.59 to -0.31) |
| Algeria | 2041409.67 (1561388.18 to 2522328.87) | 18942.18 (12008.68 to 26719.09) | 1665590.83 (1224131.71 to 2213297.39) | 13894.01 (8136.76 to 21721.92) | -0.98  (-1.01 to -0.95) |
| Cook Islands | 1170.24 (916.07 to 1476.43) | 17377.09 (11095.17 to 24649.15) | 547.67 (410.11 to 726.23) | 13738.41 (8200.36 to 20739.05) | -0.75  (-0.79 to -0.7) |
| Malaysia | 1206878.14 (1060541.66 to 1340945.72) | 18132.93 (14693.44 to 21673.19) | 1025686.8 (780102.49 to 1309426.34) | 13580.17 (8538.35 to 19854.35) | -1.01  (-1.03 to -0.98) |
| Libya | 326511.98 (255818.43 to 411756.41) | 17002.18 (10527.86 to 24586.58) | 181301.71 (130794.78 to 240375.95) | 13399.82 (7656.49 to 20870.55) | -0.93  (-1.05 to -0.81) |
| Mongolia | 185680.58 (157771.55 to 215382.71) | 20295.24 (14988.01 to 25840.15) | 136780.47 (108002.94 to 168122.72) | 13088.57 (8354.87 to 18571.92) | -1.9  (-2.07 to -1.74) |
| El Salvador | 299153.15 (231117.23 to 372985.56) | 14167.62 (9268.63 to 19960.77) | 219828.56 (165582.34 to 279352.11) | 13003.39 (8191.97 to 18961.48) | 0.07  (-0.09 to 0.23) |
| Argentina | 1885593.81 (1586465.89 to 2237244.99) | 18902.13 (13884 to 24896.37) | 1322648.8 (989187.73 to 1690384.44) | 12933.64 (7987.31 to 19514.48) | -1.59  (-1.86 to -1.32) |
| Paraguay | 274214.11 (217365.02 to 334435.33) | 16055.12 (10377.24 to 22468.24) | 241605.89 (179044.01 to 312151.83) | 12623.37 (7666.93 to 19221.01) | -0.7  (-0.77 to -0.63) |
| Brazil | 8537429.82 (6997117.97 to 10312057.62) | 16568.77 (11633.7 to 22551.66) | 5847362.86 (4596983.65 to 7314701.58) | 12532.74 (8215.45 to 17832.96) | -0.91  (-0.97 to -0.85) |
| Azerbaijan | 469297.7 (391835.22 to 555865.37) | 18934.35 (13311.36 to 25191.91) | 286447.65 (229151.99 to 353955.84) | 12502.07 (8524.91 to 17468.53) | -1.54  (-1.71 to -1.37) |
| Democratic People's Republic of Korea | 1229754.91 (1017755.47 to 1463326.23) | 17755.89 (12203.35 to 24176.81) | 603328.09 (460734.94 to 765455.52) | 12494.47 (7782.4 to 18382.85) | -1.45  (-1.64 to -1.26) |
| American Samoa | 2935.63 (2298.12 to 3627.65) | 14449.85 (9715.44 to 20131.85) | 1921.61 (1430.41 to 2444.7) | 12443.11 (7837.04 to 18268.26) | -0.48  (-0.58 to -0.38) |
| Iraq | 1390951.24 (1094582.98 to 1740640.97) | 16285.34 (11218.37 to 22703.99) | 1678783.66 (1243036 to 2207038.53) | 12383.82 (7481.74 to 18755.75) | -1  (-1.07 to -0.94) |
| Dominica | 3580.54 (2759.71 to 4509) | 14770.6 (9207.66 to 21438.13) | 1683.89 (1264.65 to 2174.17) | 12369.37 (7579.91 to 18442.56) | -0.65  (-0.79 to -0.52) |
| United Arab Emirates | 98166.18 (73653.66 to 125118.88) | 15930.68 (9826.3 to 23759.76) | 136163.66 (95491.47 to 183368.96) | 12232.57 (6623.63 to 19272.82) | -0.73  (-0.82 to -0.63) |
| Palestine | 155318.45 (120912.8 to 192547.95) | 14810.62 (9319.25 to 21269.5) | 224513.04 (171540.47 to 275308.71) | 12196.44 (7911.87 to 17635.83) | -0.67  (-0.75 to -0.58) |
| Romania | 818093.9 (665940.38 to 1001940.99) | 15368.11 (11142.93 to 20705.67) | 345950.27 (254815.34 to 445878.57) | 12168.04 (7182.1 to 18551.3) | -0.89  (-0.96 to -0.81) |
| Albania | 172079.71 (131841.14 to 214414.8) | 15294.44 (9447.31 to 22139.27) | 56889.18 (45118.67 to 72451.91) | 12131.02 (8042.92 to 17464.84) | -0.73  (-0.95 to -0.51) |
| Uruguay | 125441.55 (92857.31 to 162263.62) | 15767.42 (9640.14 to 23371.99) | 80150.49 (56360.89 to 109618.67) | 11894.17 (7026.7 to 18697.15) | -1.1  (-1.2 to -1) |
| Turkey | 4162295.91 (3270818.35 to 5073671.69) | 19325.32 (12626.8 to 26976.02) | 1739801.67 (1225359.12 to 2350760.43) | 11675.38 (6591.19 to 18494.31) | -1.93  (-2.05 to -1.81) |
| Bahrain | 30286.9 (23676.8 to 37575.57) | 17853.95 (11589.89 to 25279.24) | 25199.02 (17779.78 to 33826.14) | 11349.89 (6250.29 to 18482.02) | -1.62  (-1.68 to -1.56) |
| Kuwait | 87994.45 (75582.68 to 100967.96) | 15282.03 (11536.88 to 19601.99) | 96995.03 (71350.58 to 126260.17) | 11245.52 (6744.46 to 17063.23) | -1.1  (-1.2 to -1.01) |
| Puerto Rico | 146182.48 (107396.62 to 192646.52) | 15182.39 (8770.44 to 23463.19) | 51342.88 (34126.24 to 72698.33) | 11114.84 (5638.6 to 18566.21) | -1.23  (-1.35 to -1.12) |
| Jordan | 268116.36 (211109.07 to 331022.62) | 15821.03 (10499 to 22249.58) | 398784.64 (303716.71 to 505082.77) | 11030.52 (7000.75 to 16215.48) | -1.3  (-1.35 to -1.26) |
| South Africa | 1986830.3 (1593558.98 to 2423881.09) | 15042.96 (9903.3 to 20680.79) | 1652627.77 (1275295.81 to 2106699.59) | 11010.29 (6978.9 to 15950.96) | -1.17  (-1.48 to -0.86) |
| Brunei Darussalam | 13977.67 (10834.23 to 17486.09) | 14986.29 (9739.97 to 21554.57) | 10272.88 (7218.17 to 14150.26) | 11002.12 (6132.66 to 18203.31) | -1.06  (-1.15 to -0.97) |
| Dominican Republic | 455662.65 (359662.89 to 557508.14) | 16287.75 (10552.16 to 23172.48) | 332829.33 (250296.6 to 424627.46) | 10829.78 (6584.61 to 16488.45) | -1.22  (-1.38 to -1.07) |
| Bosnia and Herzegovina | 156952.87 (119280.85 to 197186.6) | 14581.95 (8965.61 to 21283.68) | 49131.68 (34313.67 to 65584.79) | 10816.64 (5895.33 to 17147.02) | -1.28  (-1.45 to -1.11) |
| Bulgaria | 206888.77 (151589.87 to 274888.22) | 12558.09 (7413.12 to 19546.97) | 103029.47 (72518.87 to 137770.01) | 10809.88 (5940.39 to 17033.65) | -0.56  (-0.58 to -0.54) |
| Philippines | 3955274.52 (3128174.5 to 4846369) | 15230.26 (10400.47 to 20944.75) | 3789244.83 (2962915.82 to 4674053.13) | 10577.41 (7150.48 to 14727.39) | -1.61  (-1.78 to -1.43) |
| North Macedonia | 74311.49 (59632.6 to 90078.69) | 14143.2 (9730.91 to 19134.33) | 35357.83 (27859.65 to 44628.71) | 10474.92 (6822.86 to 15133.47) | -1.06  (-1.12 to -0.99) |
| Bermuda | 1864.98 (1366.71 to 2419.13) | 15497.4 (8923.36 to 23994.09) | 849.04 (563.35 to 1193.68) | 10300.08 (5211.29 to 16997.15) | -1.45  (-1.59 to -1.3) |
| Indonesia | 15747841.91 (13652148.3 to 18280664.72) | 23323.82 (17854.64 to 29171.82) | 6466558.5 (5146871.57 to 7850537) | 10187.37 (6859.13 to 14301.07) | -3.4  (-3.7 to -3.1) |
| Republic of Moldova | 197167.97 (148554.01 to 247009.77) | 16036.75 (10152.17 to 23178.74) | 54331.47 (39851.93 to 70741.21) | 9918.47 (5918.01 to 15077.63) | -1.87  (-2.02 to -1.72) |
| Panama | 93394.53 (71378.6 to 120492.64) | 11244.61 (6969.71 to 16817.06) | 111245.65 (79461.38 to 154388.56) | 9863.16 (5497.8 to 15885.19) | -0.22  (-0.39 to -0.04) |
| Poland | 1285855.18 (965687.82 to 1642900.93) | 14055.39 (8557.43 to 20685.83) | 555294.05 (389656.28 to 765401.97) | 9701.52 (5267.16 to 15782.68) | -1.35  (-1.46 to -1.25) |
| Serbia | 260837.66 (195221.14 to 337006.47) | 12951.39 (7874.45 to 19247.12) | 130491.57 (90512.11 to 181214.24) | 9359.37 (4856.41 to 15393.15) | -1.26  (-1.34 to -1.18) |
| New Zealand | 88942.22 (64802.84 to 118574.57) | 11214.77 (7064.18 to 17065.31) | 79811.33 (52590.95 to 115801.84) | 9326.1 (4793.81 to 16239.03) | -0.62  (-0.65 to -0.6) |
| Montenegro | 17698.57 (12866.66 to 23563.19) | 11255.04 (6406.2 to 17583.74) | 9739.53 (6774.62 to 13455.98) | 9320.08 (5011.55 to 15548.27) | -0.8  (-0.88 to -0.72) |
| Lebanon | 218646.34 (175082.5 to 265136.89) | 16958.51 (11285.06 to 23584.05) | 131875.74 (85784.12 to 186675.81) | 9262.48 (4637.26 to 16227.15) | -2.18  (-2.24 to -2.12) |
| Thailand | 2214955.11 (1935274.74 to 2479830.58) | 13759.11 (11102.15 to 16747.06) | 871627.81 (588969.03 to 1233900.14) | 9076.79 (5039.21 to 15156.77) | -1.57  (-1.64 to -1.5) |
| Sri Lanka | 1063311.49 (890063.8 to 1254352.64) | 19317.55 (14457.56 to 24931.06) | 444408.69 (326601.11 to 598831.51) | 9016.62 (5095.98 to 14436.01) | -2.99  (-3.18 to -2.8) |
| Hungary | 245026.73 (180058.74 to 323245.47) | 12511.31 (7500.87 to 19073.09) | 116755.21 (80057.79 to 162168.44) | 8819.88 (4614.73 to 14942) | -1.19  (-1.32 to -1.05) |
| Slovakia | 148418.44 (108615.78 to 198691.92) | 11787.9 (6592.53 to 18259.93) | 73180.57 (49009.08 to 102021.13) | 8761.19 (4630.8 to 14605.85) | -0.99  (-1.05 to -0.94) |
| Viet Nam | 4660814.62 (3819461.43 to 5568249.38) | 17688.29 (12553.2 to 23251.61) | 1786616.43 (1316632.3 to 2304641.8) | 8624.29 (5245.66 to 13187.52) | -2.78  (-2.9 to -2.66) |
| Tunisia | 394152.43 (328274.8 to 462251.8) | 12226.25 (8963.56 to 16081.55) | 210797.75 (148460.64 to 281819.17) | 8246.68 (4721.78 to 13356.01) | -1.32  (-1.35 to -1.29) |
| Iran (Islamic Republic of) | 3857455.48 (3012461.67 to 4699231.82) | 14647.59 (9786.36 to 20106.83) | 1671796.8 (1214913.99 to 2212843.77) | 8193.9 (5023.03 to 12656.99) | -1.9  (-1.96 to -1.84) |
| Mexico | 3827102.91 (3631126.47 to 4039029.51) | 11445.39 (10413.6 to 12439.87) | 2547922.73 (2416807.04 to 2693629.68) | 8151.58 (7467.04 to 8904.76) | -0.99  (-1.18 to -0.8) |
| Qatar | 19182.29 (14964.54 to 24039.89) | 14761.3 (9534.65 to 21189.33) | 33342.55 (21866.39 to 48033.92) | 8086.71 (3991.15 to 14590.67) | -2.38  (-2.53 to -2.23) |
| Costa Rica | 132626.71 (105453.68 to 162664.82) | 11633.43 (7866.75 to 16438.06) | 82306.64 (55895.79 to 115885.19) | 8076.53 (4059.53 to 13524.77) | -1.28  (-1.37 to -1.18) |
| Czechia | 228527.17 (162295.59 to 300031.13) | 11282.22 (6534.94 to 17662.94) | 130200.57 (89272.95 to 182977.78) | 7822.74 (3971.46 to 13236.15) | -1.16  (-1.32 to -1) |
| Venezuela (Bolivarian Republic of) | 720067.39 (542885.92 to 936721.28) | 10012.31 (6086.66 to 14872.11) | 501326.05 (339928.7 to 690385.59) | 7400.58 (4027.23 to 12264.77) | -0.93  (-1.04 to -0.82) |
| Saudi Arabia | 709260.66 (594479.42 to 838590.61) | 10132.67 (7567.54 to 13076.16) | 492490.85 (328860.78 to 692517.48) | 7325.3 (4084.19 to 12113.53) | -0.92  (-1.01 to -0.82) |
| Slovenia | 40223.03 (28143.86 to 55351.14) | 10423.6 (5838.95 to 16856.13) | 21728.67 (14163.2 to 31015.15) | 7187.11 (3607.02 to 12561.61) | -1.49  (-1.58 to -1.41) |
| Lithuania | 72849.28 (49753.13 to 100221.48) | 8823.01 (4819.84 to 14356.02) | 28991.55 (19218.44 to 42064.6) | 7014.04 (3622.74 to 12429.73) | -0.82  (-0.9 to -0.73) |
| Latvia | 53676.37 (36966.42 to 71875.78) | 9312.15 (5171.41 to 14810.79) | 20417.56 (12989.15 to 29645.63) | 6810.53 (3420.26 to 11919.2) | -1.28  (-1.37 to -1.2) |
| Australia | 347945.75 (229302.91 to 502188.28) | 9461.23 (4866.97 to 16235.2) | 298970.22 (178175.5 to 464176.04) | 6733.89 (3228.86 to 12643.82) | -1.28  (-1.38 to -1.19) |
| Republic of Korea | 1381955.21 (1140094.91 to 1652781.19) | 12562.05 (8965.81 to 16995.58) | 444644.08 (309077.81 to 621229.03) | 6731.95 (3645.91 to 11329.18) | -2.06  (-2.24 to -1.88) |
| Spain | 693204.81 (449025.37 to 994674.22) | 10238.43 (5197.6 to 17149.55) | 411825.25 (240601.33 to 647033.75) | 6630.02 (2915.73 to 12973.86) | -1.57  (-1.69 to -1.45) |
| Greenland | 1401.19 (986 to 1967.79) | 9575.42 (4881.98 to 16269.95) | 758.67 (488.76 to 1105.16) | 6543.42 (3015.83 to 12015.9) | -1.19  (-1.24 to -1.13) |
| Colombia | 1554003.66 (1184316.44 to 1954342.32) | 13065.98 (8318.15 to 18939.04) | 711933.1 (494211.5 to 972177.09) | 6526.85 (3830.59 to 10325.36) | -2.52  (-2.64 to -2.41) |
| Croatia | 82145.25 (57938.66 to 111248.55) | 8911.76 (5004.75 to 14225.22) | 36332.99 (23711.36 to 53028.21) | 6464.45 (3353.78 to 11140.91) | -1.23  (-1.3 to -1.15) |
| Belarus | 221428.17 (151348.55 to 300854.98) | 9323.46 (4977.54 to 15241.13) | 104294.41 (67690.64 to 154436.1) | 6378.01 (3114.76 to 11402.56) | -1.38  (-1.57 to -1.2) |
| Japan | 1966612.1 (1357861.87 to 2696548.46) | 9021.97 (4672.09 to 15065.38) | 925999.47 (604049.16 to 1355263.63) | 6178.47 (2857.39 to 11110.75) | -1.47  (-1.56 to -1.39) |
| Nicaragua | 196148.44 (148868.89 to 253853.9) | 10501.44 (6564.57 to 15730.8) | 113299.16 (75964.87 to 155828.24) | 5797.11 (3072.27 to 10025.87) | -2.22  (-2.29 to -2.15) |
| Estonia | 31544.77 (21624.54 to 43150.19) | 9073.27 (5000.8 to 14708.49) | 11926 (7570.38 to 17716.22) | 5786.19 (2919.82 to 10470.65) | -1.88  (-1.99 to -1.76) |
| Ecuador | 709781.55 (568766.67 to 872098.63) | 18161.05 (12274.35 to 25036.33) | 282716.62 (211931.03 to 365260.53) | 5685.00 (3472.33 to 8622.14) | -4.52  (-4.71 to -4.32) |
| United Kingdom | 816728.73 (628987.63 to 1039439.82) | 7458.17 (4866.3 to 10909.76) | 652861.06 (434473.82 to 959435.16) | 5655.73 (3023.57 to 9716.66) | -0.99  (-1.05 to -0.92) |
| Singapore | 65422.92 (45866.63 to 89833.97) | 10406.01 (5626.37 to 17149.2) | 45333.04 (28738.95 to 68431.96) | 5641.07 (2635.84 to 10624.37) | -2.36  (-2.46 to -2.25) |
| Taiwan (Province of China) | 492366.21 (356231.73 to 648152.77) | 9531.55 (5493.63 to 14817.9) | 161167.71 (103758.49 to 231433.79) | 5514.66 (2777.95 to 9662.84) | -1.94  (-2.15 to -1.74) |
| Ukraine | 794786.76 (557282.76 to 1071694.63) | 7197.92 (3983.4 to 11541.69) | 348773.08 (225854.57 to 503716.69) | 5349.71 (2752.23 to 9315.94) | -1.43  (-1.57 to -1.29) |
| Israel | 124252.52 (79143.6 to 183045.91) | 8212.91 (3959.59 to 14644.64) | 141147.25 (83015.54 to 220700.83) | 5332.9 (2274.27 to 10411.53) | -1.61  (-1.71 to -1.52) |
| Russian Federation | 2279561.31 (1743022.18 to 2901012) | 6661.05 (4133.72 to 9914.04) | 1230962.93 (778375.79 to 1827797.58) | 4604.15 (2294.17 to 8221.28) | -1.63  (-1.84 to -1.42) |
| Malta | 6042.13 (3739.02 to 9468.99) | 7167.19 (3199.04 to 13913.46) | 2775.19 (1529.82 to 4488.21) | 4432.8 (1803.01 to 9218.53) | -1.73  (-1.82 to -1.64) |
| Portugal | 159231.02 (100802.45 to 241251.43) | 8395.7 (3821.23 to 15430.49) | 56439.98 (31223.29 to 93783.53) | 4409.47 (1726.43 to 9386.49) | -2.31  (-2.44 to -2.18) |
| Denmark | 58846.46 (36102.8 to 90737.87) | 7003.14 (3140.83 to 13643.22) | 37676.13 (20473.25 to 63742.9) | 4114.98 (1570.16 to 8872.05) | -2.07  (-2.2 to -1.93) |
| Greece | 102138.9 (61709.98 to 159876.42) | 5618.76 (2512.59 to 11263.34) | 55140.31 (30615.29 to 88011.55) | 4032.16 (1555.04 to 8459.66) | -1.26  (-1.43 to -1.09) |
| Finland | 66591.61 (37800.61 to 106989.37) | 7213.16 (3105.33 to 14340.2) | 31978.57 (15354.91 to 61497.82) | 4000.2 (1430.6 to 9774.26) | -2.11  (-2.27 to -1.96) |
| Norway | 51003.37 (30555.69 to 81279.05) | 6473.38 (2853.04 to 12276.61) | 35046.68 (18178.36 to 61722.8) | 3944.72 (1510.7 to 8939.9) | -1.8  (-1.96 to -1.65) |
| United States of America | 2232061.75 (1764688.91 to 2722417.52) | 4005.29 (2675.9 to 5717.12) | 2210981.78 (1557619.97 to 2991505.01) | 3781.42 (2066.19 to 6176.02) | -0.01  (-0.43 to 0.42) |
| Belgium | 107146.36 (61859.79 to 164501.53) | 6121.87 (2648.16 to 11772.59) | 68840.14 (38023.45 to 117094.99) | 3716.64 (1496.33 to 7817.99) | -1.85  (-1.94 to -1.75) |
| Austria | 78511.13 (46579.56 to 123622.24) | 5931.37 (2629.17 to 11318.07) | 47120.22 (25933.6 to 77959.26) | 3715.54 (1413.86 to 7899.45) | -1.6  (-1.72 to -1.47) |
| Sweden | 80469.83 (47584 to 123790.16) | 5157.65 (2213.93 to 9980.82) | 62863.7 (31589.52 to 108624.13) | 3577.9 (1392.14 to 7829.54) | -1.16  (-1.26 to -1.05) |
| Andorra | 462.9 (268.22 to 713.96) | 5405.82 (2295.43 to 10497.77) | 328.24 (180.15 to 564.51) | 3553.82 (1408.64 to 7856.41) | -1.52  (-1.72 to -1.32) |
| France | 679225.19 (412797.98 to 1070665.9) | 5944.32 (2687.16 to 11439.64) | 392328.6 (207673.29 to 682049.76) | 3543.17 (1409.71 to 7979.58) | -1.98  (-2.11 to -1.86) |
| San Marino | 218.18 (119.59 to 345.25) | 5139.56 (2158.84 to 10029.18) | 171.65 (92.47 to 287.19) | 3458.51 (1337.85 to 7484.94) | -1.51  (-1.64 to -1.39) |
| Luxembourg | 4175.02 (2558.9 to 6325.13) | 6350.04 (2793.7 to 12208.5) | 3306 (1829.34 to 5744.14) | 3447.11 (1345.92 to 7507.04) | -2.21  (-2.41 to -2.01) |
| Switzerland | 59780.87 (34221.76 to 96830.8) | 5233.6 (2263.5 to 10442.52) | 44404.57 (23859.51 to 73419.23) | 3430.7 (1371.44 to 7196.4) | -1.47  (-1.63 to -1.31) |
| China | 34435077.61 (31390576.63 to 37548552.53) | 10604.68 (9006.75 to 12325.94) | 7791005.7 (6422547.74 to 9395682.25) | 3415.32 (2478.93 to 4529.54) | -4.26  (-4.51 to -4.01) |
| Italy | 401222.19 (242866.09 to 622273.01) | 4763.15 (2138.17 to 9282) | 244230.52 (134175.16 to 416145.42) | 3311.53 (1330.05 to 7053.28) | -1.43  (-1.53 to -1.33) |
| Monaco | 163.03 (93.13 to 269.33) | 4735.62 (2032.43 to 9950.17) | 157.15 (83.29 to 278.28) | 3270.25 (1302.19 to 7289.36) | -1.39  (-1.51 to -1.28) |
| Germany | 751570.99 (508346.75 to 1093192.3) | 5822.71 (2851.94 to 10270.99) | 372599.19 (218340.99 to 590687.73) | 3248.23 (1344.07 to 6670.93) | -1.84  (-2.2 to -1.48) |
| Iceland | 2991.99 (1749.1 to 4791.14) | 4818.22 (2095.9 to 9682.18) | 2066.37 (1108.76 to 3616.3) | 3226.88 (1274.02 to 7099.18) | -1.49  (-1.69 to -1.29) |
| Ireland | 53564.01 (32769.99 to 84658.16) | 6033.82 (2855.28 to 11396.69) | 30584.48 (16380.56 to 54217.74) | 3187.3 (1255.02 to 7104.05) | -2.45  (-2.59 to -2.31) |
| Netherlands | 133921.43 (75789.82 to 224265.67) | 4993.37 (2197.06 to 9979.99) | 81844.86 (41467.36 to 149817.78) | 3171.73 (1253.89 to 7212.07) | -1.8  (-1.92 to -1.68) |
| Cyprus | 12335.71 (7646.29 to 18278.22) | 6400.64 (2981.82 to 11705.94) | 6223.38 (3410.08 to 10763.89) | 2923.56 (1140.98 to 6509.95) | -3.06  (-3.25 to -2.87) |
| Canada | 169533 (108082.33 to 244785.41) | 2986.71 (1465.29 to 5368.47) | 118975.65 (70049.42 to 183082.09) | 2023.79 (833.01 to 4157.10) | -1.3  (-1.45 to -1.15) |
| Chile | 156455.35 (99312.62 to 238551.94) | 3864.85 (1778.45 to 7758.28) | 54034.93 (28930.29 to 94043) | 1543.07 (600.89 to 3446.53) | -3.05  (-3.31 to -2.79) |

**Supplementary Table 7** DALY of ID among children in 1990 and 2019 and the EAPC from 1990 to 2019 in 204 countries and territories

| Location | 1990 | |  | | 2019 | |  | | 1990-2019 | |
| --- | --- | --- | --- | --- | --- | --- | --- | --- | --- | --- |
|  | DALY cases NO.(95%UI) | | ASR/100,000 (95% CI) | | DALY cases NO.(95%UI) | | ASR/100,000 (95% CI) | | EAPC (95%CI) | |
| Bhutan | 5627.35 (3017.73 to 8879.84) | | 2211 (1167.42 to 3525.36) | | 4402.15 (2886.18 to 6467.52) | | 2302.58 (1405.02 to 3478.33) | | -0.10 (-0.25 to 0.05) | |
| Mali | 69006.03 (44101.79 to 101118.56) | | 1642.06 (985.68 to 2497) | | 224232.33 (144255.85 to 324025.89) | | 2151.80 (1316.27 to 3221.81) | | 0.98  (0.76 to 1.19) | |
| Burkina Faso | 56779.32 (36464.9 to 85392.25) | | 1150.14 (635.05 to 1864.62) | | 191750.42 (118581.16 to 290045.97) | | 1801.44 (1055.54 to 2822.90) | | 1.78  (1.66 to 1.9) | |
| Gambia | 9185.72 (6069.32 to 13510.67) | | 1906.17 (1174.63 to 2860.38) | | 16519.26 (10539.94 to 24286.16) | | 1786.04 (1085.92 to 2727.81) | | -0.59  (-0.73 to -0.45) | |
| Zambia | 60545.48 (39128.49 to 87521.65) | | 1623.2 (979.92 to 2475.11) | | 133251.57 (88197.23 to 194587.4) | | 1729.27 (1055.27 to 2624.32) | | 0.36  (0.26 to 0.47) | |
| Nigeria | 648339.97 (428422.84 to 934448.18) | | 1617.84 (1020.56 to 2421.24) | | 1454920.31 (954216.97 to 2151901.87) | | 1547.54 (935.71 to 2383.87) | | -0.17  (-0.25 to -0.1) | |
| Senegal | 66144.39 (42506.73 to 97161.03) | | 1781.62 (1073.19 to 2700.24) | | 94075.28 (60669.16 to 137313.23) | | 1540.69 (940.59 to 2314.65) | | -0.62  (-0.8 to -0.44) | |
| Yemen | 81453.51 (52318.04 to 118283.59) | | 1032.4 (633.39 to 1575.57) | | 193651.37 (128184.1 to 286051.38) | | 1505.18 (948.52 to 2287.4) | | 1.71  (1.53 to 1.88) | |
| Guinea-Bissau | 5404.88 (3355.4 to 8070.96) | | 1099.52 (606.08 to 1818.35) | | 11043.94 (6906.81 to 16282.91) | | 1377.37 (783.08 to 2191.08) | | 1.07  (0.88 to 1.27) | |
| Sierra Leone | 18011.84 (10347.74 to 27990.89) | | 1110.7 (580.07 to 1821.61) | | 45992.64 (29286.77 to 69308.06) | | 1369.97 (811.18 to 2156.7) | | 0.8  (0.74 to 0.86) | |
| Chad | 40442.44 (24837.04 to 60469.66) | | 1327.32 (750.67 to 2134.64) | | 116253.9 (74950.07 to 174717.89) | | 1363.83 (779.2 to 2159.66) | | 0.03  (-0.04 to 0.09) | |
| Niger | 57773.73 (36729.98 to 88408.68) | | 1385.91 (787.86 to 2180.22) | | 160372.99 (99020.34 to 236966.31) | | 1315.33 (755.5 to 2077.8) | | -0.33  (-0.52 to -0.14) | |
| Mauritania | 14776.73 (9589.23 to 21781.92) | | 1527.28 (919.72 to 2359.11) | | 20400.09 (11095.54 to 31941.43) | | 1275.74 (636.54 to 2115.49) | | -0.39  (-0.46 to -0.32) | |
| Togo | 19004.7 (11651 to 29685.83) | | 1039.51 (569.95 to 1717.22) | | 40060.75 (25503.89 to 60302.56) | | 1265.37 (742.72 to 1989.86) | | 0.67  (0.4 to 0.94) | |
| India | 4898369.76 (3320352.47 to 6997486.02) | | 1468.89 (979.64 to 2107.53) | | 4685688.81 (3134824.9 to 6782302.17) | | 1257.19 (829.35 to 1827.72) | | -0.51  (-0.58 to -0.45) | |
| Malawi | 59191.28 (37661.14 to 88551.51) | | 1318.73 (774.16 to 2063.34) | | 93063.1 (58027.78 to 137848.38) | | 1188.51 (698.1 to 1851.89) | | -0.61  (-0.78 to -0.43) | |
| Pakistan | 660686.58 (409567.22 to 979591.31) | | 1292.02 (731.11 to 2022.38) | | 1032352.86 (633025.15 to 1565116.37) | | 1185.3 (660.87 to 1899.36) | | -0.49  (-0.67 to -0.31) | |
| Haiti | 25789.6 (16060.3 to 38471.08) | | 951.86 (535.38 to 1522.54) | | 49723.81 (31572.1 to 76431.77) | | 1160.99 (668.07 to 1860.08) | | 0.88  (0.82 to 0.93) | |
| United Republic of Tanzania | 183184.26 (120114.42 to 265492.79) | | 1484.7 (928.25 to 2235.32) | | 286281 (181352.92 to 427114.09) | | 1159.93 (658.19 to 1817.95) | | -1.08  (-1.25 to -0.9) | |
| Somalia | 44227.1 (28185.49 to 64601.14) | | 1270.66 (731.46 to 1964.73) | | 107119.98 (68246.48 to 160284.91) | | 1114.12 (648.17 to 1770.65) | | -0.55  (-0.59 to -0.5) | |
| Guinea | 27146.68 (17130.88 to 40872) | | 882.54 (497.83 to 1410.72) | | 64794.57 (40710.98 to 95265.66) | | 1105.48 (641.92 to 1754.61) | | 0.89  (0.78 to 1) | |
| Mozambique | 73010.19 (46563.72 to 109167.96) | | 1173.92 (672 to 1858.75) | | 154199.07 (99398.22 to 231665.31) | | 1098.34 (649.69 to 1727.74) | | -0.15  (-0.21 to -0.09) | |
| Eritrea | 19918.75 (12643.9 to 29887.43) | | 1400.61 (821.39 to 2216.14) | | 28596.15 (17560.66 to 42173.56) | | 1081.75 (595.13 to 1730.9) | | -1.01  (-1.07 to -0.96) | |
| South Sudan | 30674.03 (18699.11 to 45565.2) | | 1166.71 (644.76 to 1853) | | 45110.5 (27218.43 to 68057.76) | | 1080.52 (574 to 1764.12) | | -0.39  (-0.46 to -0.33) | |
| Comoros | 2749.34 (1270.8 to 4263.6) | | 1288.76 (572.47 to 2117.85) | | 2351.75 (1322.6 to 3583.39) | | 1016.14 (512.44 to 1693.36) | | -1.09  (-1.25 to -0.93) | |
| Gabon | 4598.06 (2832.94 to 7122.33) | | 1095.38 (582.12 to 1796.95) | | 5888.85 (3462.6 to 8919.45) | | 1013.27 (530.55 to 1630.87) | | -0.13  (-0.4 to 0.14) | |
| Central African Republic | 10948.76 (6037.64 to 17473.05) | | 858.33 (406.03 to 1491.76) | | 22889.14 (12677.62 to 37672.71) | | 1011.16 (480 to 1737.35) | | 0.42  (0.31 to 0.53) | |
| Ghana | 62398.27 (39076.77 to 94220.35) | | 863.26 (479.38 to 1415.68) | | 113226.36 (68470.41 to 171419.76) | | 1001.39 (560.54 to 1633.62) | | 0.57  (0.41 to 0.74) | |
| Cabo Verde | 1889.01 (1189.55 to 2844.29) | | 1189.14 (667.27 to 1894.49) | | 1566.37 (926.17 to 2332.27) | | 995.14 (525.79 to 1658.99) | | -0.72  (-0.82 to -0.63) | |
| Djibouti | 2759.56 (1699.74 to 4119.4) | | 1243.98 (699.6 to 1975.77) | | 4031.21 (2391.81 to 6320.33) | | 947.19 (483.52 to 1562.34) | | -1.14  (-1.28 to -1) | |
| Myanmar | 166946.78 (101344.31 to 256732.31) | | 1050.43 (571.1 to 1730.74) | | 138934.09 (85902.36 to 214527.14) | | 942.92 (521.42 to 1556.41) | | -0.36  (-0.4 to -0.32) | |
| Democratic Republic of the Congo | 184859.75 (116559.45 to 277732.76) | | 998.5 (580.06 to 1592.14) | | 351511.68 (218676.49 to 521946.93) | | 927.81 (527.73 to 1469.9) | | -0.32  (-0.5 to -0.13) | |
| Ethiopia | 276166.17 (181271.04 to 397599.09) | | 1076.35 (682.75 to 1575.49) | | 427927.1 (282653.05 to 627553.88) | | 920.39 (588.3 to 1388.96) | | -0.63  (-0.68 to -0.58) | |
| Benin | 19817.84 (11984.51 to 30295.16) | | 776.63 (423.33 to 1270.69) | | 51738.73 (32532.85 to 77723.56) | | 886.19 (490.24 to 1452.3) | | 0.18  (-0.1 to 0.47) | |
| Congo | 9493.94 (5766.19 to 14353.12) | | 861.9 (461.16 to 1421.83) | | 17202.07 (9953.47 to 27095.26) | | 861.5 (435.3 to 1439.85) | | 0.04  (-0.06 to 0.13) | |
| Lesotho | 5745.21 (3467.14 to 8726.98) | | 754.22 (409.34 to 1231.16) | | 5398.21 (3402.44 to 8216.33) | | 829.14 (475.45 to 1331.31) | | 0.51  (0.37 to 0.64) | |
| Burundi | 23612.33 (14581.45 to 36219.69) | | 861.77 (453.2 to 1426.42) | | 45561.11 (28496.23 to 67522.99) | | 827.03 (467.02 to 1344.68) | | 0.4  (0.21 to 0.59) | |
| Nepal | 65936.17 (40620.09 to 102630.62) | | 745.61 (411.74 to 1250.23) | | 73779.89 (47109.22 to 118512.3) | | 816.65 (445.67 to 1347.15) | | 0.04  (-0.11 to 0.18) | |
| Cameroon | 36450.95 (23352.18 to 54267.27) | | 727.07 (416.64 to 1172.93) | | 97150.32 (58397.98 to 152318.92) | | 805.19 (437.54 to 1327.37) | | 0.4  (0.2 to 0.59) | |
| Cambodia | 48844.38 (30263.19 to 73997.53) | | 991.38 (554.17 to 1614.37) | | 40175.05 (25031.83 to 61538) | | 797.74 (440.03 to 1341.84) | | -0.72  (-0.83 to -0.62) | |
| Uzbekistan | 87637.47 (54745.07 to 131938.26) | | 1001.4 (557.27 to 1626.05) | | 82580.89 (49291.89 to 125654.54) | | 797.42 (405.94 to 1326.87) | | -0.83  (-0.94 to -0.73) | |
| Papua New Guinea | 14460.42 (8855.19 to 21629.82) | | 849.21 (465.34 to 1356.78) | | 29953.12 (18096.29 to 44690.27) | | 794.92 (438.44 to 1296.93) | | -0.05  (-0.11 to 0.01) | |
| Sao Tome and Principe | 468.21 (175.87 to 771.41) | | 811.14 (263.05 to 1497.34) | | 575.21 (321.67 to 920.34) | | 794.17 (366.45 to 1440.2) | | 0.68  (-0.32 to 1.69) | |
| Madagascar | 62941.02 (40350.64 to 93108.47) | | 1106.51 (636.45 to 1757.98) | | 85138.91 (51997.09 to 129898.06) | | 776.81 (428.74 to 1268.8) | | -1.39  (-1.66 to -1.12) | |
| Namibia | 6064.83 (3251.71 to 9520.92) | | 1010.54 (492.86 to 1674.1) | | 6255.24 (3760 to 9656.18) | | 744.51 (395.53 to 1246.66) | | -1.11  (-1.3 to -0.93) | |
| Marshall Islands | 204.2 (128.71 to 300.04) | | 906.29 (539.01 to 1402.89) | | 131.76 (80.43 to 202.61) | | 730.07 (401.23 to 1212.58) | | -0.79  (-0.84 to -0.74) | |
| Kiribati | 254.66 (160.63 to 381.98) | | 840.02 (461.86 to 1342.9) | | 304.63 (181.62 to 473.13) | | 724.61 (383.55 to 1232.11) | | -0.5  (-0.54 to -0.45) | |
| Uganda | 90014.44 (57854.08 to 133437.82) | | 1025.13 (586.97 to 1613.26) | | 136736.1 (84328.85 to 213990.66) | | 710.47 (384.39 to 1167.26) | | -1.25  (-1.48 to -1.02) | |
| Equatorial Guinea | 2054.9 (1237.46 to 3261.76) | | 981.8 (517.31 to 1635.21) | | 3907.36 (2213.99 to 6328.08) | | 701.52 (330.07 to 1230.94) | | -1.44  (-1.61 to -1.26) | |
| Sudan | 84479.91 (51801.1 to 126792.37) | | 900.39 (497.94 to 1449.87) | | 109437.66 (65951.04 to 168087.92) | | 693.67 (374.56 to 1152.46) | | -0.7  (-0.76 to -0.64) | |
| Botswana | 5258.63 (3303.65 to 7877.07) | | 917.49 (529.72 to 1463.63) | | 4829.42 (2918.73 to 7424.93) | | 685.56 (348.24 to 1192.96) | | -0.97  (-1.1 to -0.85) | |
| Fiji | 1881.96 (1173.53 to 2859.49) | | 669.4 (374.67 to 1095.89) | | 1811.6 (1080.92 to 2823.46) | | 684.01 (346.31 to 1157.55) | | 0.3  (0.18 to 0.42) | |
| Solomon Islands | 1221.84 (746.77 to 1869.93) | | 760.41 (406.33 to 1263.01) | | 1790.01 (1068.56 to 2752.35) | | 683.24 (356.22 to 1147.4) | | -0.3  (-0.37 to -0.24) | |
| Liberia | 8501.82 (5104.97 to 12968.73) | | 914.95 (488.33 to 1532.65) | | 12575.88 (6937.54 to 19592.98) | | 679.19 (331.06 to 1149.37) | | -0.86  (-0.95 to -0.76) | |
| Bolivia (Plurinational State of) | 21406.44 (13574.98 to 31828.87) | | 784.46 (439.89 to 1242.55) | | 27236.5 (16845.07 to 42566.28) | | 672.62 (363.28 to 1108.24) | | -0.14  (-0.28 to 0.01) | |
| Guyana | 2472.07 (1490.52 to 3815.66) | | 871.98 (453.42 to 1474.41) | | 1392.35 (790.22 to 2126.96) | | 670.17 (334.37 to 1159) | | -0.6  (-0.69 to -0.51) | |
| Angola | 28615.63 (16966.74 to 44228.38) | | 580.33 (297.18 to 956.02) | | 94717.41 (56120.48 to 143724.58) | | 665.09 (339.71 to 1121.16) | | 0.8  (0.69 to 0.92) | |
| Zimbabwe | 25106.14 (14576.68 to 39572.49) | | 514.14 (252.2 to 889.55) | | 39264.63 (23286.56 to 59561.51) | | 656.16 (351.18 to 1103.62) | | 1.19  (0.87 to 1.51) | |
| Timor-Leste | 2419.22 (715.96 to 4152.89) | | 686.34 (170.34 to 1274.01) | | 3212.36 (1980 to 4935.61) | | 640.32 (337.38 to 1098.44) | | -0.7  (-0.89 to -0.5) | |
| Grenada | 255.02 (163 to 375.86) | | 797.11 (482.05 to 1251.08) | | 126.94 (73.16 to 200.21) | | 590.44 (290.16 to 1065.39) | | -0.88  (-0.99 to -0.78) | |
| Bangladesh | 549690.69 (340565.59 to 831567.27) | | 1097.41 (609.16 to 1762.23) | | 249952.7 (147585.79 to 406729.55) | | 586.95 (293.87 to 1034.3) | | -2.38  (-2.48 to -2.27) | |
| Suriname | 903.81 (515.98 to 1403.47) | | 689.7 (337.03 to 1202.62) | | 827.21 (477.62 to 1316.66) | | 572.6 (267.58 to 1046.93) | | -0.58  (-0.61 to -0.55) | |
| Micronesia (Federated States of) | 388.61 (251.3 to 585.87) | | 818.41 (479.11 to 1286.51) | | 169.98 (102.56 to 260.77) | | 570.52 (287.65 to 971.69) | | -1.23  (-1.33 to -1.14) | |
| Belize | 548.22 (319.98 to 860.65) | | 674.53 (339.73 to 1155.38) | | 680.06 (404.04 to 1071.92) | | 569.52 (270.28 to 1018.79) | | -0.56  (-0.64 to -0.49) | |
| Rwanda | 27035.72 (16647.67 to 39505.24) | | 772.67 (425.91 to 1264.97) | | 27346.82 (16538.89 to 41690.74) | | 567.7 (302.31 to 950.92) | | -1.06  (-1.29 to -0.82) | |
| Tuvalu | 26.03 (15.89 to 39.62) | | 753.83 (398.21 to 1275.03) | | 18.57 (10.78 to 29.22) | | 562.68 (277.89 to 980.55) | | -0.88  (-0.96 to -0.81) | |
| Eswatini | 2378.36 (1399.75 to 3724.44) | | 607 (302.93 to 1055.79) | | 2271.02 (1347.04 to 3540.55) | | 557.04 (277.67 to 959.94) | | -0.14  (-0.33 to 0.05) | |
| Saint Vincent and the Grenadines | 253.71 (145.59 to 403.9) | | 615.37 (285.34 to 1103.54) | | 132.76 (73.6 to 210.87) | | 543.22 (239.5 to 1003.8) | | -0.44  (-0.54 to -0.35) | |
| Kyrgyzstan | 12327.95 (7515.35 to 18362.17) | | 725.12 (392.37 to 1194.56) | | 11263.33 (6715.04 to 17706.51) | | 530 (267.99 to 920.58) | | -1.2  (-1.32 to -1.08) | |
| Mauritius | 2626.75 (1648.88 to 3977.85) | | 834.06 (470.21 to 1369.13) | | 1021.38 (605.28 to 1645.78) | | 518.6 (262.45 to 930.11) | | -1.52  (-1.61 to -1.44) | |
| Nauru | 29.89 (18.19 to 46.35) | | 634.94 (330.2 to 1120.07) | | 19.67 (11.36 to 31.94) | | 498.54 (241.57 to 899.05) | | -0.66  (-0.83 to -0.48) | |
| Vanuatu | 276.84 (164.52 to 441.69) | | 397.02 (196.45 to 703.57) | | 545.96 (320.72 to 831.02) | | 494.91 (245.71 to 856.02) | | 0.42  (0.25 to 0.58) | |
| Samoa | 377.33 (229.07 to 596.44) | | 586.92 (299.59 to 1023.86) | | 342.34 (195.26 to 560.35) | | 487.64 (234.14 to 874.64) | | -0.5  (-0.62 to -0.37) | |
| Tokelau | 5.27 (3.24 to 8.14) | | 711.5 (363.24 to 1227.82) | | 2.32 (1.39 to 3.64) | | 481.3 (228.83 to 848.63) | | -1.34  (-1.36 to -1.31) | |
| Saint Lucia | 342.78 (197.29 to 552.32) | | 654.81 (316.6 to 1149.54) | | 147.53 (82.69 to 247.39) | | 481.3 (209.19 to 914.07) | | -1.03  (-1.14 to -0.91) | |
| Guatemala | 20742.03 (12692.03 to 31812.97) | | 556.03 (300.99 to 910.97) | | 27800.88 (16898.91 to 42612.18) | | 481.1 (245.08 to 836.57) | | -0.24  (-0.51 to 0.02) | |
| Trinidad and Tobago | 2553.87 (1448.51 to 4103.64) | | 630.47 (297.25 to 1150.29) | | 1273.84 (680.35 to 2111.37) | | 477.74 (203.84 to 904.03) | | -1.09  (-1.19 to -0.99) | |
| Lao People's Democratic Republic | 13879.62 (8400.56 to 21551.67) | | 749.75 (396.69 to 1281.84) | | 10731.22 (6305.1 to 17079.66) | | 476.99 (226.27 to 844.85) | | -1.73  (-1.82 to -1.63) | |
| Afghanistan | 28211.89 (16962.3 to 43939.37) | | 557.8 (285.36 to 951.79) | | 83599.64 (49709.23 to 130851.71) | | 473.8 (238.73 to 800.56) | | -0.52  (-0.59 to -0.44) | |
| Barbados | 410.98 (258.16 to 629.94) | | 647.77 (363.02 to 1060.13) | | 232.68 (132.16 to 375.24) | | 465.8 (220.08 to 853.5) | | -1.13  (-1.21 to -1.05) | |
| Kazakhstan | 35437.79 (22566.13 to 53097.7) | | 681.14 (386.26 to 1088.48) | | 23642.35 (13994.31 to 37526.19) | | 458.64 (226.65 to 815.35) | | -1.69  (-1.83 to -1.55) | |
| Antigua and Barbuda | 120.99 (73.18 to 188.57) | | 648.09 (313.71 to 1150.06) | | 73.84 (42.48 to 119.97) | | 456.85 (200.33 to 855.61) | | -1.34  (-1.43 to -1.26) | |
| Jamaica | 4596.56 (2707.87 to 7123.79) | | 561.15 (287.14 to 967.01) | | 2616.49 (1426.74 to 4289.94) | | 450.89 (204.86 to 832.4) | | -0.81  (-0.97 to -0.65) | |
| Bahamas | 444.42 (248.39 to 728.47) | | 558.65 (256.74 to 1027.06) | | 341.52 (186.07 to 545.89) | | 448.76 (195.79 to 864.89) | | -0.72  (-0.86 to -0.57) | |
| Morocco | 64156.68 (38278.58 to 99765.07) | | 650.78 (333.25 to 1122.22) | | 40687.34 (22798.44 to 67481.7) | | 440.45 (198.78 to 829.32) | | -1.2  (-1.26 to -1.14) | |
| Tajikistan | 14409.59 (8080.57 to 22524.08) | | 604.28 (302.41 to 1035.76) | | 14453.1 (8379.13 to 22550.82) | | 440.05 (215.16 to 774.31) | | -1.34  (-1.44 to -1.24) | |
| Armenia | | 5370.35 (3215.03 to 8259.8) | | 509.73 (258.31 to 855.24) | | 2593.34 (1510.22 to 4163.32) | | 427.03 (215.59 to 755.3) | | -0.47  (-0.57 to -0.37) |
| Oman | | 8434.13 (5345.78 to 12894.09) | | 971 (580.13 to 1505.23) | | 4531.95 (2359.28 to 7645.39) | | 419.07 (173.64 to 814.51) | | -2.81  (-3.08 to -2.54) |
| Peru | | 68730.59 (42967.38 to 105468.57) | | 815.54 (448.29 to 1348.94) | | 37612.47 (21354.52 to 61443) | | 414.14 (198.57 to 756.97) | | -2.6  (-2.78 to -2.42) |
| Georgia | | 7148.02 (4130.25 to 11406.88) | | 525.71 (254.4 to 926.41) | | 2995.44 (1655.38 to 4920.66) | | 411.61 (184.78 to 756.13) | | -0.7  (-0.81 to -0.6) |
| Maldives | | 1328.24 (817.81 to 1948.78) | | 1207.11 (678.64 to 1924.53) | | 467.06 (259.23 to 753.51) | | 411.53 (185.45 to 771.9) | | -4.36  (-4.61 to -4.11) |
| Guam | | 206.03 (115.73 to 329.96) | | 476.74 (226.5 to 864.34) | | 184.83 (99.81 to 299.93) | | 406.63 (178.54 to 752.1) | | -0.25  (-0.48 to -0.01) |
| Egypt | | 156761.85 (92679.74 to 256215.44) | | 701.58 (362.08 to 1226.95) | | 132945.48 (73030.07 to 218940.7) | | 403.92 (184.12 to 749.08) | | -1.62  (-1.82 to -1.42) |
| Turkmenistan | | 9264.57 (5790.68 to 14211.56) | | 600.68 (314.09 to 985.31) | | 6200.26 (3706.51 to 9720.05) | | 402.26 (191.54 to 710.45) | | -1.54  (-1.64 to -1.43) |
| Kenya | | 58560.13 (38400.32 to 85387.65) | | 505.36 (329.53 to 749.56) | | 75712.11 (49744.17 to 111416.36) | | 399.99 (258.16 to 592.94) | | -0.87  (-0.99 to -0.75) |
| Mongolia | | 6751.79 (4152.63 to 10215.79) | | 744.42 (417.07 to 1197.07) | | 4041.87 (2287.19 to 6271.48) | | 394.8 (187.41 to 695.18) | | -2.74  (-2.98 to -2.5) |
| Tonga | | 185.39 (112.12 to 291.11) | | 475.14 (240.15 to 824.66) | | 139.92 (82.59 to 232.22) | | 394.74 (187.28 to 731.91) | | -0.55  (-0.62 to -0.49) |
| Honduras | | 12412.32 (7640.42 to 18944.04) | | 554.84 (299.26 to 917.92) | | 12735.44 (7449.63 to 20669.72) | | 389.34 (191.02 to 693.51) | | -1.09  (-1.16 to -1.03) |
| Palau | | 24.35 (14.19 to 38.62) | | 518.36 (249.08 to 925.88) | | 12.1 (6.84 to 19.86) | | 384.08 (169 to 733.88) | | -0.9  (-1.02 to -0.79) |
| Niue | | 4.71 (2.57 to 7.48) | | 566.61 (275.8 to 1007.12) | | 1.47 (0.77 to 2.43) | | 382.62 (165.24 to 719.96) | | -1.41  (-1.5 to -1.32) |
| Malaysia | | 42587.24 (27702.91 to 63360.53) | | 642.79 (390.25 to 979.42) | | 28799.51 (16506.73 to 45453.87) | | 379.05 (176.53 to 688) | | -1.85  (-1.9 to -1.8) |
| United States Virgin Islands | | 157.86 (88.98 to 253.23) | | 501 (230.81 to 931.49) | | 73.52 (39.79 to 125.22) | | 374.33 (156.7 to 741.88) | | -1.12  (-1.32 to -0.93) |
| Brazil | | 293036.47 (178250.47 to 447967.09) | | 565.8 (298.42 to 948.63) | | 175507.72 (105983.54 to 272736.55) | | 373.93 (190.46 to 657.29) | | -1.35  (-1.44 to -1.27) |
| Saint Kitts and Nevis | | 73.43 (40.36 to 118.69) | | 527.09 (239.25 to 951.47) | | 42.59 (23.31 to 69.58) | | 371.59 (155.63 to 711.76) | | -1.14  (-1.27 to -1.01) |
| Syrian Arab Republic | | 35928.33 (21250.24 to 58030.8) | | 579.13 (289.35 to 1031.05) | | 13226.45 (7263.53 to 22064.6) | | 356.66 (153.63 to 691.36) | | -1.78  (-1.98 to -1.57) |
| Algeria | | 60256.39 (34276.83 to 94673.06) | | 558.43 (262.82 to 985.19) | | 41563.66 (23123.43 to 72111.97) | | 348.31 (152 to 695.19) | | -1.48  (-1.53 to -1.43) |
| Paraguay | | 8623.31 (5126.61 to 13755.54) | | 506.87 (255.91 to 890.21) | | 6711.29 (3813.72 to 10656.22) | | 348.1 (162.37 to 643.5) | | -1.12  (-1.22 to -1.02) |
| Northern Mariana Islands | | 59.11 (33.55 to 96.09) | | 457.76 (208.11 to 834) | | 23.51 (12.59 to 39.13) | | 342.77 (151.73 to 661.57) | | -0.66  (-0.87 to -0.45) |
| Cook Islands | | 32.32 (18.27 to 51.61) | | 480.98 (220.5 to 881.35) | | 13.46 (7.57 to 22.42) | | 335.36 (148.44 to 647.39) | | -1.15  (-1.21 to -1.09) |
| Libya | | 9193.72 (5130.69 to 15176.81) | | 481.81 (220.3 to 876.03) | | 4623.14 (2483.96 to 8141.49) | | 335.16 (143.82 to 663.72) | | -1.4  (-1.57 to -1.23) |
| Cuba | | 11274.35 (6206.04 to 18823.69) | | 452.85 (196.81 to 866.45) | | 5804.66 (3012.87 to 9999) | | 326.18 (128.76 to 653.25) | | -1.16  (-1.27 to -1.05) |
| American Samoa | | 81.9 (47.17 to 129.46) | | 407.43 (199.59 to 714.25) | | 50.63 (29.43 to 82.4) | | 324.9 (151.01 to 603.75) | | -0.72  (-0.86 to -0.59) |
| Seychelles | | 129.09 (76.4 to 209.22) | | 545.6 (274.14 to 963.18) | | 70.27 (37.57 to 115.82) | | 324.65 (137.08 to 633.03) | | -1.85  (-1.94 to -1.76) |
| South Africa | | 60314.15 (36384.3 to 93752.01) | | 455.91 (226.48 to 788.1) | | 48617.19 (28671.58 to 77402.13) | | 322.2 (155.16 to 569.74) | | -1.27  (-1.63 to -0.9) |
| El Salvador | | 8436.77 (4906.98 to 13708.24) | | 400.4 (193.99 to 716.59) | | 5371.18 (2929.88 to 8886.88) | | 315.18 (143.94 to 582.68) | | -0.41  (-0.59 to -0.23) |
| Democratic People's Republic of Korea | | 31449.73 (18158.53 to 49958.93) | | 472.84 (234.16 to 821.68) | | 15255.34 (8550.25 to 24693.82) | | 315.08 (133.42 to 584.13) | | -1.64  (-1.9 to -1.39) |
| Dominica | | 95.63 (55.26 to 156.41) | | 392.6 (179.42 to 733.25) | | 42.9 (24.19 to 71.78) | | 306.86 (135.17 to 579.3) | | -0.9  (-1.08 to -0.72) |
| Iraq | | 39658.33 (22981.91 to 62983.89) | | 467.98 (238.06 to 824.25) | | 41517.26 (22163.83 to 70350.12) | | 305.38 (136.36 to 577.23) | | -1.56  (-1.68 to -1.45) |
| Azerbaijan | | 13735.78 (8238.67 to 21517.18) | | 558.96 (291.55 to 964.44) | | 7029.91 (4029.82 to 11367.15) | | 303.03 (148.96 to 545.35) | | -2.33  (-2.56 to -2.11) |
| Palestine | | 4260.08 (2529.49 to 6779.48) | | 415.04 (196.65 to 736.71) | | 5451.06 (3108.89 to 8498) | | 294.22 (138.58 to 539.07) | | -1.16  (-1.29 to -1.03) |
| North Macedonia | | 2445.35 (1488.93 to 3839.57) | | 462.43 (242.9 to 808.96) | | 996.71 (569.85 to 1590.58) | | 293.12 (136.71 to 535.19) | | -1.62  (-1.71 to -1.53) |
| Romania | | 23026.11 (13227.62 to 35628.51) | | 429.75 (219.43 to 753.96) | | 8394.42 (4637.42 to 13799.76) | | 291.36 (126.41 to 566.66) | | -1.45  (-1.53 to -1.37) |
| United Arab Emirates | | 2588.7 (1420.59 to 4190.4) | | 425.79 (189.88 to 796.61) | | 3341.29 (1756.23 to 5696.72) | | 290.04 (114.35 to 566.4) | | -1.06  (-1.19 to -0.92) |
| Albania | | 4880.2 (2811.71 to 7845.22) | | 435 (204.57 to 786.56) | | 1351.43 (792.27 to 2247.71) | | 287.85 (135.07 to 539.39) | | -1.34  (-1.59 to -1.09) |
| Argentina | | 51721.51 (31159.57 to 82065.6) | | 517.84 (276.78 to 884.56) | | 29437.74 (16836.32 to 48739.53) | | 286.02 (136.58 to 550.86) | | -2.35  (-2.7 to -1.99) |
| Jordan | | 8053.88 (4671.31 to 12892.66) | | 479.5 (233.89 to 836.41) | | 10099.72 (5729.6 to 16446.8) | | 276.03 (123.8 to 519.54) | | -2.01  (-2.07 to -1.95) |
| Indonesia | | 504805.13 (319275.47 to 780361.04) | | 743.55 (429.29 to 1186.22) | | 179156.09 (104741.42 to 281330.73) | | 275.59 (141.33 to 477.76) | | -4.02  (-4.33 to -3.71) |
| Kuwait | | 2426.06 (1464.89 to 3655.79) | | 425.9 (232.93 to 686.41) | | 2348.89 (1336.11 to 3885.3) | | 271.84 (123.55 to 529.7) | | -1.6  (-1.72 to -1.49) |
| Turkey | | 124710.74 (73035.41 to 193053.14) | | 579.97 (280.48 to 996.36) | | 40993.17 (22420.23 to 71476.78) | | 271.6 (110.76 to 545.59) | | -2.89  (-3.05 to -2.73) |
| Dominican Republic | | 13141.48 (7665.53 to 21210.96) | | 475.53 (229.98 to 858.61) | | 8061.94 (4363.82 to 13285.86) | | 263.65 (116.25 to 506.84) | | -1.77  (-1.97 to -1.56) |
| Philippines | | 112024.94 (66589.8 to 175343.55) | | 434.49 (222.16 to 752.35) | | 94109.43 (56007.19 to 154199.34) | | 263.53 (131.67 to 479.88) | | -2.08  (-2.26 to -1.9) |
| Bosnia and Herzegovina | | 4556.63 (2596.63 to 7377.86) | | 419.8 (192.57 to 763.01) | | 1217.64 (681.94 to 2074.35) | | 263.13 (105.66 to 523.35) | | -2  (-2.25 to -1.76) |
| Bulgaria | | 5596.89 (3029.37 to 9235.1) | | 335.46 (147.73 to 643.37) | | 2539.22 (1342.34 to 4145.33) | | 262.83 (105.88 to 510.27) | | -0.93  (-0.97 to -0.89) |
| Bahrain | | 859.79 (496.84 to 1413.4) | | 510.91 (248.43 to 920.81) | | 592.58 (316.16 to 1021.27) | | 261.95 (107.01 to 535.88) | | -2.4  (-2.47 to -2.32) |
| Brunei Darussalam | | 375.9 (219.87 to 581.74) | | 409.24 (205.17 to 717.02) | | 243.32 (134.91 to 412.45) | | 258.05 (107.99 to 512.2) | | -1.61  (-1.74 to -1.48) |
| Uruguay | | 2999.35 (1647.46 to 5041.32) | | 375.67 (166.21 to 715.55) | | 1644.38 (857.66 to 2842.67) | | 242.64 (101.67 to 487.99) | | -1.66  (-1.8 to -1.52) |
| Puerto Rico | | 3651.44 (1927.76 to 6025.06) | | 374.88 (157.49 to 712.51) | | 1132.43 (574.29 to 1975.64) | | 232.88 (86.43 to 480.41) | | -1.87  (-2.02 to -1.72) |
| Poland | | 37354.9 (21162.42 to 60961.59) | | 401.2 (186.1 to 722.83) | | 13383.15 (6970.25 to 23395.49) | | 231.16 (94.4 to 464.85) | | -2.02  (-2.16 to -1.88) |
| Republic of Moldova | | 4954.93 (2936.18 to 8087.95) | | 402.38 (192.12 to 751.29) | | 1234.2 (666.51 to 2146.19) | | 219.1 (92.85 to 431.86) | | -2.35  (-2.51 to -2.19) |
| Serbia | | 7296.52 (4041.91 to 11624.28) | | 357.35 (164.53 to 674.77) | | 3095.7 (1613.3 to 5449.54) | | 217.92 (86.56 to 455.08) | | -1.92  (-2.03 to -1.81) |
| Viet Nam | | 151284.03 (91037.65 to 231675.56) | | 573.11 (307.59 to 953.64) | | 44515.01 (24023.01 to 75041.18) | | 213.22 (96.69 to 399.9) | | -3.77  (-3.92 to -3.62) |
| Panama | | 2321.4 (1225.04 to 3758.02) | | 278.82 (122.41 to 518.7) | | 2411.25 (1262.22 to 4111.51) | | 212.46 (83.91 to 421.94) | | -0.58  (-0.77 to -0.39) |
| Montenegro | | 450.6 (246.86 to 749.36) | | 283.98 (117.82 to 557.21) | | 225.05 (119.04 to 378.17) | | 212.34 (84.85 to 436.23) | | -1.24  (-1.37 to -1.12) |
| Bermuda | | 46.3 (25.16 to 80.13) | | 386.98 (163.43 to 770.13) | | 17.79 (9.06 to 31.49) | | 210.29 (75.39 to 434.01) | | -2.15  (-2.33 to -1.96) |
| Mexico | | 116679.97 (77233.94 to 170766.17) | | 349.4 (226.56 to 518.75) | | 66274.56 (43390.7 to 97516.3) | | 209.76 (134.81 to 312.78) | | -1.53  (-1.77 to -1.29) |
| Sri Lanka | | 32029.51 (19834.33 to 49488.32) | | 578.26 (319.7 to 946.16) | | 10395.78 (5492.38 to 17905.26) | | 206.69 (85.62 to 409.02) | | -3.94  (-4.13 to -3.75) |
| Hungary | | 6594.35 (3605.85 to 10741.56) | | 330.12 (145.51 to 621.19) | | 2675.46 (1369.26 to 4742.21) | | 199.32 (77.55 to 418.42) | | -1.73  (-1.91 to -1.55) |
| Slovakia | | 3907.49 (2107.73 to 6687.79) | | 305.58 (130.99 to 593.3) | | 1642.21 (825.44 to 2862.8) | | 195.4 (76.46 to 401.19) | | -1.52  (-1.6 to -1.44) |
| Iran (Islamic Republic of) | | 107589.94 (63497.93 to 160149.96) | | 408.19 (200.86 to 698.06) | | 38805.93 (21381.73 to 66771.89) | | 189.87 (83.46 to 364.24) | | -2.43  (-2.53 to -2.33) |
| Tunisia | | 10123.83 (6296.01 to 15470.25) | | 314.66 (165.68 to 539.21) | | 4562.54 (2342.45 to 7723.98) | | 177.1 (74.89 to 352.11) | | -1.91  (-1.95 to -1.87) |
| Thailand | | 54628.49 (33332.44 to 82410.81) | | 336.73 (194.15 to 542.37) | | 17296.96 (8997.84 to 31027.7) | | 176.59 (73.62 to 367.64) | | -2.37  (-2.47 to -2.27) |
| Costa Rica | | 3286.93 (1868.67 to 5367.11) | | 289.32 (144.06 to 523.33) | | 1816.5 (922.46 to 3162.97) | | 176.19 (66.38 to 361.72) | | -1.74  (-1.87 to -1.61) |
| Lebanon | | 5259.65 (3122.85 to 8183) | | 415.53 (208.22 to 731.74) | | 2479.63 (1248.35 to 4546.77) | | 175.42 (65.7 to 378.38) | | -3.06  (-3.15 to -2.97) |
| Republic of Korea | | 43797.65 (26429.83 to 68079.7) | | 392.82 (210.34 to 656.18) | | 11348.95 (6164.31 to 19047.02) | | 170.21 (69.95 to 339.54) | | -2.76  (-3 to -2.51) |
| New Zealand | | 1771.16 (958.34 to 3147.06) | | 224.08 (98.19 to 441.21) | | 1440.49 (699.19 to 2669.1) | | 167.35 (60.7 to 389.48) | | -0.98  (-1.01 to -0.96) |
| Czechia | | 5876.41 (3215.92 to 9665.69) | | 284.54 (121.56 to 551.59) | | 2800.42 (1512 to 4828.88) | | 167.1 (61.83 to 341.05) | | -1.71  (-1.92 to -1.5) |
| Venezuela (Bolivarian Republic of) | | 17851.94 (10237.24 to 29666.63) | | 249.29 (113.61 to 473.29) | | 10753.28 (5537.3 to 18159.8) | | 156.7 (63.55 to 307.71) | | -1.47  (-1.64 to -1.31) |
| Saudi Arabia | | 18059.79 (10711.48 to 27802.23) | | 259.59 (138.04 to 435.87) | | 10487.7 (5407 to 18235.23) | | 154.62 (61.52 to 313.37) | | -1.51  (-1.65 to -1.38) |
| Qatar | | 452.24 (262.92 to 735.9) | | 355.36 (175.41 to 634.17) | | 627.4 (309.99 to 1163.77) | | 151.34 (53.85 to 348.42) | | -3.34  (-3.55 to -3.14) |
| Slovenia | | 1002.18 (543.85 to 1693.51) | | 254.29 (107.9 to 520.25) | | 457.02 (234.99 to 828.61) | | 149.01 (56.89 to 321.04) | | -2.15  (-2.27 to -2.02) |
| United Kingdom | | 22271.64 (13407.32 to 33951.56) | | 203.41 (107.21 to 345.65) | | 16812.34 (9318.47 to 27735.73) | | 145.57 (66.32 to 285.1) | | -1.22  (-1.29 to -1.15) |
| Greenland | | 32.17 (17.26 to 54.06) | | 224.59 (86.67 to 464.68) | | 16.06 (7.87 to 29.53) | | 138.05 (48.54 to 304.79) | | -1.54  (-1.6 to -1.48) |
| Lithuania | | 1568.5 (827.52 to 2747.06) | | 189.74 (75.06 to 387.84) | | 562.67 (273.41 to 1000.3) | | 135.74 (50.28 to 290.85) | | -1.23  (-1.34 to -1.12) |
| Latvia | | 1184.74 (610.27 to 2059.91) | | 206.2 (84.24 to 415.23) | | 393.04 (201.08 to 704.91) | | 130.75 (50.91 to 275.94) | | -1.89  (-2.01 to -1.77) |
| Colombia | | 39224.82 (21949.85 to 63357.94) | | 331.45 (155.38 to 602.8) | | 14217.89 (7467.68 to 23902.36) | | 130.54 (54.38 to 262.04) | | -3.31  (-3.45 to -3.17) |
| Japan | | 47145.98 (24362.09 to 79115.3) | | 209.42 (84.01 to 416.72) | | 19454.12 (9690.55 to 34908.46) | | 126.43 (45.05 to 270.44) | | -1.96  (-2.08 to -1.85) |
| Croatia | | 1852.55 (957.34 to 3187.83) | | 197.61 (81.39 to 385.58) | | 708.25 (369.96 to 1253.64) | | 123.55 (47.46 to 260.52) | | -1.78  (-1.89 to -1.67) |
| Taiwan (Province of China) | | 13501.9 (7607.49 to 22354.16) | | 254.95 (113.9 to 484.17) | | 3599.58 (1763.4 to 6540.16) | | 121.68 (47.18 to 263) | | -2.62  (-2.88 to -2.37) |
| Nicaragua | | 4899.83 (2735.76 to 8067.88) | | 264.93 (116.61 to 505.47) | | 2396.06 (1141.02 to 4197.9) | | 121.04 (45.58 to 262.08) | | -2.91  (-2.99 to -2.83) |
| Ecuador | | 20438.15 (12064.99 to 32567.63) | | 524.27 (273.37 to 899.41) | | 6012.5 (3492.64 to 9850.29) | | 120.31 (57.91 to 224.17) | | -5.61  (-5.83 to -5.38) |
| Belarus | | 4953.2 (2647.58 to 8709.69) | | 207.14 (83.68 to 429.94) | | 1975.12 (989.14 to 3506.63) | | 120.22 (43.12 to 267.04) | | -2.01  (-2.25 to -1.77) |
| Spain | | 14709.81 (6844.69 to 26415.99) | | 208.43 (73.49 to 434.99) | | 7432.37 (3278.93 to 15295.9) | | 115.71 (36.79 to 287.85) | | -2.1  (-2.28 to -1.93) |
| Singapore | | 1643.02 (878.06 to 2794.02) | | 256.72 (100.45 to 503.73) | | 883.77 (438.82 to 1567.19) | | 111.56 (37.99 to 248.23) | | -3.21  (-3.37 to -3.06) |
| Australia | | 6304.51 (3110.81 to 12209.32) | | 170.59 (64.22 to 375.22) | | 4754.84 (2084.18 to 9223.01) | | 106.37 (35.95 to 250.59) | | -1.75  (-1.88 to -1.63) |
| Ukraine | | 17330.95 (9645.98 to 29275.9) | | 155.27 (62.02 to 315.65) | | 6990.76 (3524.69 to 12630.29) | | 104.64 (40.85 to 229.08) | | -1.91  (-2.09 to -1.73) |
| Estonia | | 689.65 (365.04 to 1229.85) | | 198.11 (80.8 to 415.1) | | 217.55 (111.72 to 410.59) | | 104 (39.31 to 236.12) | | -2.7  (-2.86 to -2.54) |
| Israel | | 2606.81 (1221.95 to 4965.55) | | 171.2 (60.37 to 372.49) | | 2561.72 (1170.32 to 5201.26) | | 97.66 (30.92 to 226.94) | | -2.09  (-2.21 to -1.96) |
| Russian Federation | | 51852.83 (29922.32 to 82466.88) | | 150.29 (68.93 to 278.34) | | 24571.76 (12536.6 to 43968.5) | | 91.16 (33.69 to 193.52) | | -2.21  (-2.47 to -1.95) |
| China | | 1066246.55 (678797.03 to 1566549.96) | | 329.15 (197.67 to 503.45) | | 187278.87 (115365.61 to 291199.15) | | 82.6 (43.25 to 138.21) | | -5.14  (-5.43 to -4.84) |
| Portugal | | 3319.97 (1571.11 to 6377.6) | | 169.51 (58.75 to 394.2) | | 965.66 (399.48 to 2118.14) | | 73.4 (20.79 to 200.62) | | -3  (-3.17 to -2.82) |
| United States of America | | 42019.16 (24543.52 to 66414.65) | | 75.57 (38.78 to 134.14) | | 43331.78 (22916.17 to 75348.67) | | 72.43 (29.98 to 142.19) | | 0.02  (-0.42 to 0.45) |
| Malta | | 114.21 (53.49 to 220.31) | | 133.99 (46.78 to 320.46) | | 45.31 (19.87 to 92.18) | | 72.31 (21.63 to 176.87) | | -2.24  (-2.35 to -2.13) |
| Greece | | 1880.23 (828.81 to 3796.63) | | 99.33 (32.63 to 240.28) | | 917.81 (381.08 to 1945.39) | | 65.72 (19.17 to 171.75) | | -1.58  (-1.79 to -1.37) |
| Denmark | | 1044.35 (473.75 to 1979.22) | | 124.03 (39.2 to 293.3) | | 597.43 (265.59 to 1242.06) | | 64.43 (18.5 to 162.45) | | -2.57  (-2.73 to -2.4) |
| Norway | | 863.14 (399.13 to 1734.8) | | 109.86 (37.06 to 252.64) | | 548 (222.91 to 1227.7) | | 60.37 (16.51 to 170.91) | | -2.18  (-2.37 to -1.98) |
| Austria | | 1399.37 (641.57 to 2759.25) | | 104.69 (33.88 to 244.66) | | 741.12 (323.24 to 1465.09) | | 58.34 (17.34 to 147.94) | | -2.03  (-2.19 to -1.87) |
| Belgium | | 1896.53 (919.06 to 3583.07) | | 107.57 (37 to 248.75) | | 1091.34 (448.92 to 2202.49) | | 57.84 (17.05 to 142.75) | | -2.3  (-2.41 to -2.19) |
| Sweden | | 1351.99 (650.19 to 2484.23) | | 87.74 (28.64 to 207.75) | | 1010.03 (424.02 to 2146.32) | | 56.82 (15.79 to 146.21) | | -1.42  (-1.55 to -1.29) |
| Andorra | | 8.19 (3.67 to 16.17) | | 92.59 (29.9 to 215.4) | | 5.5 (2.22 to 12.1) | | 55.68 (16.03 to 140.24) | | -1.84  (-2.07 to -1.6) |
| France | | 12088.45 (5537.21 to 22244.03) | | 104.88 (35.05 to 247.21) | | 6220.78 (2579.68 to 12917.55) | | 54.79 (15.52 to 148.63) | | -2.49  (-2.65 to -2.34) |
| Italy | | 7396.13 (3562.68 to 13369.1) | | 85.93 (29.67 to 193.9) | | 4137.33 (1862.8 to 8681.11) | | 54.33 (15.58 to 132.59) | | -1.79  (-1.93 to -1.66) |
| Luxembourg | | 74.72 (35.37 to 135.36) | | 113.55 (38.35 to 260.73) | | 52.48 (22.33 to 109) | | 54.03 (15.34 to 139.13) | | -2.68  (-2.93 to -2.43) |
| San Marino | | 3.78 (1.62 to 7.32) | | 87.39 (26.05 to 213.18) | | 2.74 (1.19 to 5.62) | | 53.82 (15.56 to 138.88) | | -1.87  (-2.02 to -1.72) |
| Germany | | 13574.83 (6947.12 to 25166.07) | | 105.05 (39.83 to 227.69) | | 6183.43 (2916.34 to 12064.69) | | 53.46 (16.82 to 130.34) | | -2.11  (-2.51 to -1.71) |
| Switzerland | | 999.01 (457.28 to 1974.98) | | 87.33 (27.65 to 217.49) | | 683.48 (288.28 to 1415.19) | | 52.55 (15.31 to 133.51) | | -1.79  (-1.97 to -1.6) |
| Finland | | 988.18 (445.51 to 1937.4) | | 105.72 (34.9 to 241.54) | | 423.67 (157.86 to 937.93) | | 51.36 (14.03 to 145.62) | | -2.62  (-2.81 to -2.44) |
| Monaco | | 2.74 (1.18 to 5.83) | | 78.97 (24.49 to 200.3) | | 2.47 (1.03 to 4.99) | | 50.68 (14.89 to 130.74) | | -1.69  (-1.83 to -1.56) |
| Iceland | | 50.48 (22.84 to 100.69) | | 80.62 (25.93 to 197.82) | | 32.49 (13.09 to 66.59) | | 49.58 (13.78 to 133.76) | | -1.85  (-2.09 to -1.6) |
| Ireland | | 935.53 (429.85 to 1867.43) | | 102.55 (35.18 to 237.06) | | 459.48 (192.35 to 958.71) | | 46.65 (13.91 to 120.89) | | -3.05  (-3.23 to -2.87) |
| Cyprus | | 237.84 (114.64 to 440.58) | | 121.81 (42.56 to 268.6) | | 98.92 (44.17 to 211.12) | | 46.5 (13.74 to 121.9) | | -3.76  (-4 to -3.53) |
| Netherlands | | 1939.24 (840.91 to 3809.05) | | 72.32 (24.35 to 172.26) | | 1074.29 (441.11 to 2404.82) | | 41.09 (10.87 to 107.24) | | -2.28  (-2.43 to -2.12) |
| Canada | | 3627.27 (1851.23 to 6398.81) | | 63.38 (22.92 to 138.02) | | 2359.18 (1127.28 to 4392.15) | | 39.29 (12.42 to 89.66) | | -1.63  (-1.8 to -1.46) |
| Chile | | 2662.91 (1316.48 to 4974.58) | | 66.64 (23.23 to 156.08) | | 837.04 (347.12 to 1766.24) | | 23.23 (6.24 to 60.4) | | -3.54  (-3.79 to -3.29) |
